# Generating Functional Multicellular Organoids from Human Placenta Villi

**DOI:** 10.1002/advs.202301565

**Published:** 2023-07-12

**Authors:** Lijun Huang, Zhaowei Tu, Liudan Wei, Wei Sun, Yifan Wang, Shilei Bi, Fang He, Lili Du, Jingsi Chen, Julia Kzhyshkowska, Haibin Wang, Dunjin Chen, Shuang Zhang

**Affiliations:** ^1^ Department of Obstetrics and Gynecology The Third Affiliated Hospital of Guangzhou Medical University Guangzhou 510150 China; ^2^ Institute of Transfusion Medicine and Immunology Medical Faculty Mannheim University of Heidelberg 68167 Mannheim Germany; ^3^ Fujian Provincial Key Laboratory of Reproductive Health Research Department of Obstetrics and Gynecology The First Affiliated Hospital of Xiamen University School of Medicine Xiamen University Xiamen 361005 China; ^4^ Key Laboratory for Major Obstetric Diseases of Guangdong Province Guangzhou 510150 China; ^5^ Guangdong‐Hong Kong‐Macao Greater Bay Area Higher Education Joint Laboratory of Maternal‐Fetal Medicine Guangzhou 510150 China; ^6^ Guangdong Engineering and Technology Research Center of Maternal‐Fetal Medicine Guangzhou 510150 China

**Keywords:** placenta villi, organoid, preeclampsia, trophoblast, immune cell

## Abstract

The interaction between trophoblasts, stroma cells, and immune cells at the maternal–fetal interface constitutes the functional units of the placenta, which is crucial for successful pregnancy outcomes. However, the investigation of this intricate interplay is restricted due to the absence of efficient experimental models. To address this challenge, a robust, reliable methodology for generating placenta villi organoids (PVOs) from early, late, or diseased pregnancies using air–liquid surface culture is developed. PVOs contain cytotrophoblasts that can self‐renew and differentiate directly, along with stromal elements that retain native immune cells. Analysis of scRNA sequencing and WES data reveals that PVOs faithfully recapitulate the cellular components and genetic alterations of the corresponding source tissue. Additionally, PVOs derived from patients with preeclampsia exhibit specific pathological features such as inflammation, antiangiogenic imbalance, and decreased syncytin expression. The PVO‐based propagation of primary placenta villi should enable a deeper investigation of placenta development and exploration of the underlying pathogenesis and therapeutics of placenta‐originated diseases.

## Graphical abstract

A robust and reliable method is developed for creating placenta villi organoids (PVOs), which faithfully replicate multiple cellular components, maintain trophoblast stemness, preserve intrinsic stromal immune cells, and offer translational applications with patient‐specific organoids. This research holds substantial value and potential impact in the placenta field.

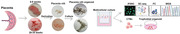

## 1 Introduction

The placenta is an extraembryonic organ that originates from the blastocyst trophectoderm and provides vital support to the fetus during intrauterine life.^[^ ^1^ ^]^ The bulk of the fetal‐maternal interface by mass is formed by the placental villi, which consists of a complex arrangement of trophoblast lineages, stromal cells, endothelial cells, and immune cells. Cytotrophoblasts, which rapidly proliferate, fuse to form multinucleated syncytiotrophoblast (STB) or migratory extravillous trophoblast (EVT).^[^ ^2^ ^]^ The STBs, which cover the entire surface of the placenta villi, secrete hormones and growth factors that regulate the maternal blood supply to the placenta and facilitate maternal‐fetal communication.^[^ ^3^ ^]^ Meanwhile, EVTs located at the tips of the anchoring villi invade into the maternal endometrium and remodel the uterine spiral arteries to ensure adequate perfusion of the placenta during pregnancy.^[^ ^4^ ^]^ The core stromal cell provides structural support for the villi and produces extracellular matrix components. Endothelial cells, which line the small blood vessels within the stroma, play an essential role in regulating the blood flow and oxygen supply to the developing fetus.^[^ ^5^ ^]^

Pregnancy success depends on a delicate balance between immune activation and embryonic antigen tolerance due to the semi‐allogeneic nature of the fetus.^[^ ^6^ ^]^ At the maternal–fetal interface, various immune cell types, including decidual natural killer (dNK) cells, macrophages, T cells, dendritic cells (DC), B cells, and NKT cells, interact with decidual stromal cells and trophoblasts, forming a complex network of cellular connections.^[^ ^7^ ^]^ However, imbalances in cellular immunity can lead to adverse pregnancy outcomes, such as miscarriage, preeclampsia, preterm birth, fetal growth restriction, and infection.^[^ ^8^ ^]^

Recently, several methods have been developed to model human placenta development using human trophoblast stem cells (hTSCs). These hTSCs can be generated from various sources, including trophoblast stem cell lines or organoids derived from blastocysts, placental villi from first‐trimester or late pregnancy tissues.^[^ ^9^ ^]^ These cell lines represent a significant advancement in isolating bona fide hTSCs. In addition, naïve human pluripotent stem cells have been induced to form hTSCs that are similar to those derived from blastocysts, thus overcoming tissue limitations.^[^ ^10^ ^]^ When cultured in 3D, naïve hPSC‐derived hTSCs self‐organize into stem‐cell‐derived trophoblast organoids with villous architecture similar to primary trophoblast organoids.^[^ ^11^ ^]^ Despite a low efficiency of TSC generation, several groups have identified enhancing factors for hTSC derivation from primed hPSC and successfully obtained hTSC‐like cells that are functionally equivalent to blastocyst‐derived hTSCs,^[^ ^12^ ^]^ providing another alternative in vitro placental model. Furthermore, trophoblast stem cells can also be induced via reprogramming of somatic cells, such as fibroblasts using Yamanaka factors OCT4, KLF4, SOX2, and c‐MYC (OKSM) and a selection of TS cell culture conditions, allowing generate patient‐specific iTS cells to interrogate the trophoblast and placenta biology as well as their interactions with embryonic cells in health and diseases.^[^ ^13^ ^]^ However, these cell models contain only trophoblast lineages and cannot reflect the complex maternal‐fetal interactions that occur during pregnancy. Therefore, the establishment of a functional placental experimental model that can faithfully recapitulate the microenvironment during pregnancy for in‐depth research the maternal‐fetal interactions is urgently needed.

Air–liquid (ALI) based organoids have been broadly used to model diseases in multiple other organs including intestine, brain and pancreas,^[^ ^14^ ^]^ as they mimic the native tissue microarchitecture. In this study, we present a versatile, reliable, cost‐and time‐effective methodology for expanding human primary placenta villi by incorporating the ALI system. This system allows for sustained villi proliferation, branching and multilineage differentiation over 30 days. The characteristics of trophoblast stem cells, stoma cells and immune cells were analyzed using single‐cell RNA sequencing and validated by IHC, IF and flow cytometry. The fidelity of genetic alterations in each paired tissues were also determined using whole genome sequencing. We also demonstrated the efficacy of this method in culturing PVs from late or diseased pregnancy of 52 patients, highlighting the potential of PVOs as a valuable tool for investigating underlying cellular/molecular mechanisms and prevention strategies of placenta‐originated disease.

## 2 Results

### 2.1 Establishment of a Long‐Term Placenta Villi Organoid (PVOs) Culture System

**Figure 1:**
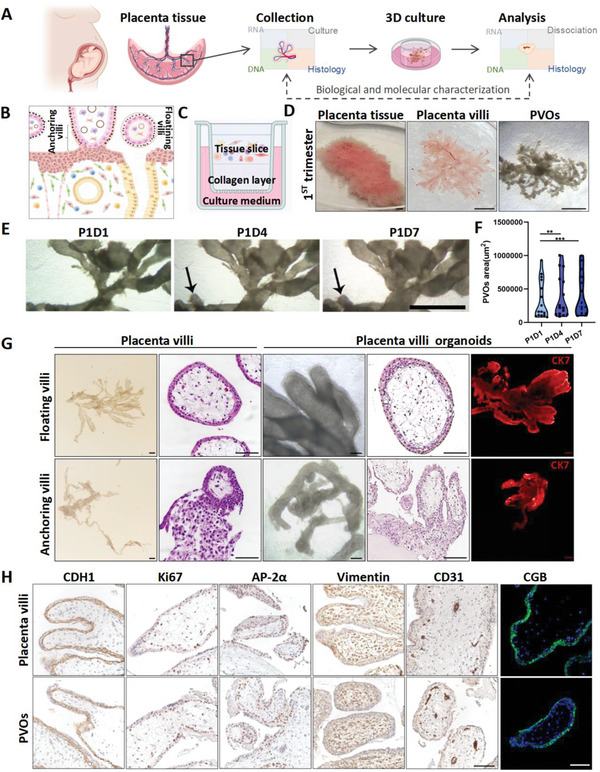
PVOs recapitulate the anatomical and histological features of the human placenta villi. A) Experimental design and workflow for PVO culture and analysis. Placenta was obtained from patients undergoing surgery (patient information detailed in Table S1 in the Supporting Information) and processed as described in the methods. B) Schematic of placenta at the maternal‐fetal interface during the first trimester, showing the anatomical position of floating and anchoring villi. C) Schematic representation of 3D culture system. Placenta villi organoids were maintained in collagen gels under an air–liquid interface microenvironment. D) The morphology of the first‐trimester placenta tissue, placenta villi, and generated PVOs at day 7. E) Representative bright‐filed pictures of individual villi during PVO culture proved their expansion. Arrows indicated branches. Scale bars, 500 µm. F) Quantification of the PVO area at P1D1, P1D4, and P1D7 of culture. ** *p* < 0.01. *** *p* < 0.001. G) Anatomical morphology and representative H&E staining of floating villi, anchoring villi, and corresponding PVOs cultured at day 7. Whole‐mount immunostaining for CK7 (trophoblast marker) in PVOs from the first trimester (6–9 weeks gestation) placenta. The experiments were repeated twice independently. Scale bars, 100 µm. H) Immunostaining of CDH1, Ki67, AP‐2α, CGB, VIM, and CD31 in the first‐trimester placenta and PVOs (cultured for 7 days). Similar results were obtained with three independent organoids. Scale bars, 100 µm.

To establish a 3D model of multicellular, functional PVOs, we collected 52 placental tissues from early or late pregnancy. All subjects were divided into four parts for histology, RNA, DNA extraction and 3D culture, respectively (**Figure** **1**A). We started to try the 3D culture of PVOs from first‐trimester placenta samples, as trophoblast cells at this stage contain more progenitor cells and were highly proliferative.^[^ ^15^ ^]^ Different from the trophoblast organoid culture, a collagen gel with an air–liquid interface was used, culture medium was optimized based on ALI medium (Figure 1A and Table S1, Supporting Information). Specifically, PGE2, an important lipid molecule that activates trophoblast stem cell proliferation,^[^ ^16^ ^]^ was included in the medium. Additionally, a selective small molecule inhibitor of Rho‐associated kinase Y‐27632, was added to increase primary human organoid formation efficiency and passaging capability. We observed that the growth of PVOs was more dependent on growth factors such as EGF and FGF2, probably due to the strong FGFR2 expression in the placenta of the 1st trimester.^[^ ^17^ ^]^ Placenta villi from the chorionic plate and decidua basalis, including floating villi and anchoring villi, were scraped and mechanically dissociated cautiously, followed by seeding on the gel of an air–liquid surface (Figure 1B,C). The culture system yielded expanding villous structures and branching as in vivo (Figure 1D‐F). The length and area of PVOs continuously increased along the in vitro culture from day 1 to day 7 (Figure 1E,F; Figure S1A,B, Supporting Information). After 7–14 days of culture, all PVOs retained the structural and cellular components of placenta villi at maternal‐fetal interface. Whole‐mount immunofluorescence staining revealed expression of a pan‐trophoblast marker, CK7, indicating the outer layer of PVOs from the 1st trimester were of epithelial origin (Figure 1G). IHC and IF staining on paraffin sections of PVOs further showed that different trophoblast subsets including SCT, marked by CGB and GCM1, and VCT, marked by TFAP2a and GATA3, were maintained in PVOs (Figure 1H; Figure S1C, Supporting Information). Notably, the polarization and lining order of SCT (outside) and VCT (inside) were well phenocopied (Figure 1H; Figure S1C, Supporting Information).

In our system, PVOs were capable of producing continuous cell sheets that facilitated coculture of epithelial cells and stroma without reconstitution. IHC staining of PVOs with the stroma marker vimentin revealed positive signals underlying trophoblast. Besides, the endothelial cells marked by CD31 were present inside the stroma layer (Figure 1H; Figure S1C, Supporting Information).

The viability of PVOs was found to decrease with prolonged culture time. While some cases (50%) showed villous structure and proliferative cells after 30 days of culture, most PVOs became sporadic with extensive vacuolation and poor microvilli beyond three passages (usually less than 30 days) (Figure S1D, Supporting Information). Despite this limitation, the culture system was effective in studying placental development and disease pathology in a range of conditions, including missed delivery (2 cases), recurrent spontaneous abortion (6 cases), preterm delivery (4 cases), and pre‐eclampsia (12 cases), although the yield was relatively lower than that obtained from PVOs derived from first‐trimester placenta (40 cases) (**Table** **1**), which is known to be enriched with trophoblast stem cells.^[^ ^18^ ^]^

**Table 1:** Summary of sample information for PVOs derivation, related to Figures 1 and 5

Sample source	Gestation	Case	Success rate
Selective abortion	6–9 weeks	40	87.5% (35)
Inevitable abortion	6–9 weeks	6	66.7 % (4)
Preterm birth	28–37 weeks	4	75% (3)
Early onset preeclampsia	28–34 weeks	12	83.3% (10)

**Figure 2:**
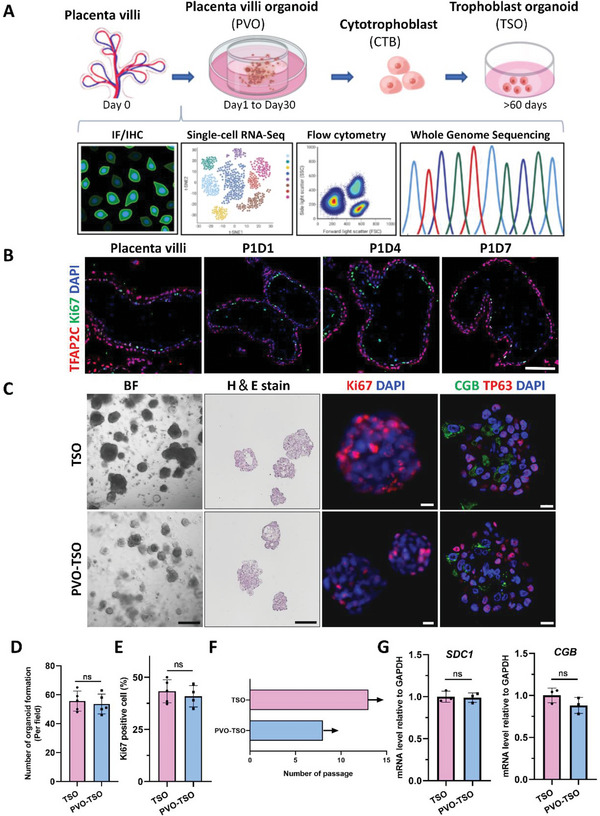
PVOs contained stem cells for long‐term trophoblast organoid culture. A) Schematic depicting the derivation, the culture of placenta villi organoids, and procedure for establishing trophoblast organoids from PVO. PVOs were digested for cytotrophoblast cells and expanded to generate conventional trophoblast organoids. They can be expanded and passages for more than 60 days. Established PVOs are validated using several methods: i) Expression of marker genes for cell populations by immunostaining, ii) Single‐cell RNA sequencing, iii) The expression profile of immune cells by flow cytometry and iv) Genetic analysis by Whole Genome Sequencing. Placental villi were used as a control. B) Immunofluorescence staining of Ki67 and TFAP2C in placenta villi and PVOs (Day 1, 4, 7). Scale bars, 50 µm. C) Images of Bright‐field, H&E stain of TSO and PVO‐TSO, Scale bars,100 µm. Confocal microscopy images of trophoblast organoid stained for Ki67, TP63. and HCG. Representative images from *n* =  3 (TSO) and *n* =  3 (PVO‐derived TSO). Scale bars, 10 µm. D) The organoid formation efficiency was assayed after 14 days of culture using a conventional trophoblast organoid medium (TOM). Organoids from 5 randomly selected microscopic fields were counted. The data were shown as means ± SD. No significant difference was observed between the groups based on unpaired *t*‐test. E) Quantification of Ki67 positive cells shown in Figure 2C. Five pictures of random fields were taken for analysis. The data were shown as means ± SD. No significant difference was observed between the groups based on unpaired *t*‐test. F) The number of passages for the organoids derived from placenta villi and PVO are shown. G) Relative mRNA expression of *CGB* and *SDC1* in TSO and PVO‐derived TSO. ns, not significant.

As floating and anchoring villi differ in their cellular composition and functionality, we detected molecular signatures and EVT differentiation in anchoring villi. As shown in Figure S1C and S2A (Supporting Information), both anchoring and floating villi retained cytotrophoblasts (CTBs), marked by GATA3 and TFAP2A, and STBs, marked by GCM1. Immunohistochemical staining for Ki67 in PVO showed that proliferative cells are mostly localized in the inner CTB layer of floating villi and CCCs of anchoring villi. HLA‐G, an EVT marker, showed specific localization of proximal CCCs of anchoring villi (Figure S2A, Supporting Information). To test the capacity of EVT differentiation, we cultured PVOs derived from floating villi and anchoring villi with EVTM. The differentiated EVT cells sprouted out of PVO and adhered to the plastic and stained positive for CK7. As depicted in revised **Figure** **2**B (Supporting Information), we observed more EVT differentiation from anchoring villi PVO, possibly due to more EVT progenitors in this structure.

### 2.2 Trophoblast Stem Cells Retained in PVOs Can be Used for Long‐Term Trophoblast Organoid Culture

To test whether the trophoblast stemness is well preserved in PVOs by seeding them as organoids, PVOs were digested, and the EpCAM^+^ cells were sorted and transferred into Matrigel droplet with normal trophoblast organoid medium (TOM), and the organoid forming capability in these cells was then checked (Figure 2A). To confirm the existence of trophoblast stem cells in the PVOs, we costained Ki67 and TFAP2C and found the retained Ki67^+^ CTBs in the organoids (Figure 2B). Notably, we observed that these cells were capable to form TSOs with similar efficiency as those derived from early primary placenta villi (Figure 2C,D). This observation was further confirmed by the comparable number of Ki67^+^ cells in the two distinct originated TSOs (Figure 2E). The PVO‐TSOs can be maintained and passaged extensively, similar to TSOs (Figure 2F). In addition, PVO‐derived TSOs expressed trophoblast markers such as TP63, SDC1, and CGB (Figure 2C,G). These findings demonstrate that the stemness of trophoblast cells was preserved in PVOs and that they represent an additional resource for TSO culture.

### 2.3 PVOs Recapitulate Molecular Signatures and Preserve the Immune Cell Components

**Figure 3:**
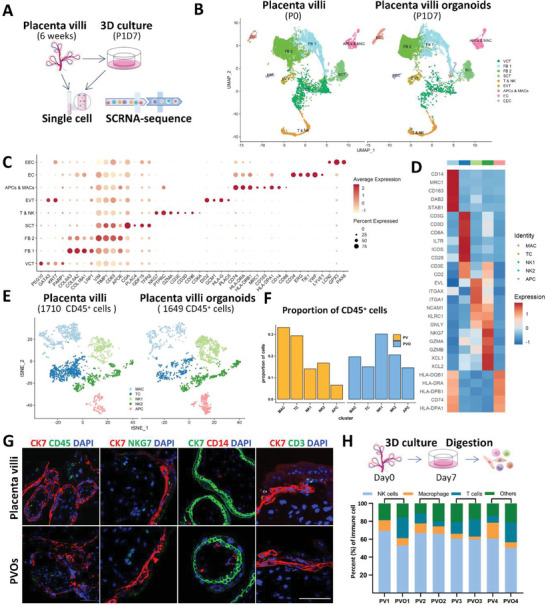
PVOs accurately phenocopy the molecular signatures and preserve the immune cell components. A) Workflow of the single‐cell RNA sequence using placenta villi and PVO. Single‐cell RNA sequencing was performed using the 10× Genomics platform followed by bioinformatics analysis. B) Placental villi and PVO cell clusters from 10× Genomics and scRNA‐seq analysis visualized by UMAP. 10 464 cells isolated from placenta villi and 10 365 cells of PVO were included (VCT, villous cytotrophoblast. FB, fibroblasts. SCT, syncytiotrophoblast. T&NK, T cells, and natural killer cells. EVT, extravillous trophoblast. APC&MAC, Antigen presenting cells and macrophages. EC, endothelial cells. EEC, endometrium epithelial cell). C) Dotplot map showing the expression of classical cell type‐specific marker genes in each cell cluster. D) Heat map showing relative expression of markers defining the subset of 5 immune cell populations. E) t‐SNE plots of CD45^+^ cells from fresh placenta villi (1710 cells, left) or day 7 PVO (1649 cells, right). F) t‐SNE plots denoting the localization of PV and PVO to 3359 CD45^+^ immune cells identified by (E). G) Representative confocal microscopy images of PV and PVO stained for CK7, CD45, NKG7, CD14, and CD3. Experiments were performed in six biological replicates. Scale Bar, 50 µm. H) Flow cytometry analysis to identify immune components in placenta villi and their derived PVOs at d7. Biological replicates, *n* = 4.

To further shed light on the cellular diversity and proportions of different cell types found in PVOs and their originated tissue, we performed scRNA‐seq on placenta villi from 6 weeks of gestation and the corresponsive PVO derived after 7 days in culture, respectively (**Figure** **3**A). Profiled cells were excluded with ≤600 genes expressed, high mitochondrial RNA content (mtRNA ≥20%), and/or potential doublets. In total, 10464 cells from placenta villi and 10 362 cells from PVOs were remained for subsequent analyses using Seurat package. Based on previously defined cell‐type markers in placenta of early pregnancy,^[^ ^19^ ^]^ we detected 9 distinct cellular populations that correspond to the key cell types found in vivo. Clusters were visualized using UMAP (Uniform Manifold Approximation and Projection) (Figure 3B) and annotated based on marker genes (Figure 3C; Table S3, Supporting Information). Consistent with the early placenta, PVOs retained a relatively proportion of villous cytotrophoblast (VCT, around 5%) in which the stem and progenitor cells mainly presented, with a similar proportion of differentiated cells including syncytiotrophoblast (13.4%), extravillous trophoblast (1.95%) (Table S4, Supporting Information). In contrast to conventional trophoblast organoids,^[^ ^9d^ ^]^ PVOs in culture also retained stroma fibroblast populations termed FB1 and FB2, as well as endothelial cells (Figure 3C). Notably, the native immune cells in maternal‐fetal interface including T and NK cells, macrophages and Antigen presenting cells (APCs), were also largely preserved (Figure 3C). As the tissues were isolated from maternal‐fetal interface of early gestation, a small portion of Endometrial Epithelial Cells (EEC) were retained both in vivo (around 1%) and in vitro (around 5%). Overall, these analyses demonstrate that PVO faithfully recapitulated the cellular components of its source tissue.

The immune cells that reside at the maternal‐fetal interface are thought to play vital roles in pregnancy,^[^ ^20^ ^]^ we therefore reanalyzed the CD45^+^ population, which was further classified into five subpopulations, including NK cells (NK1, NK2), T cells, macrophages, and antigen presenting cells based on well‐defined markers (Figure 3D). All immune subclusters presented in PVOs exhibited a similar profiling pattern to the originating placenta villi (PV) (Figure 3E). Analysis of immune cell proportions reflected that the levels of APCs and NK cells were comparable in PVOs to those in placenta villi, whereas macrophages and T cells were reduced in PVOs (Figure 3F; Table S4, Supporting Information).

To confirm the immune cell compositions reflected in scRNA‐seq, we compared the immune repertoire of the same placenta tissue and its derived PVOs using flow cytometry (Figure S3A, Supporting Information). In line with the scRNA‐seq data, FACS analysis revealed a comparable percentage of natural killer cells marked by CD56 and lymphoid lineage marked by CD3^+^ cells in both PVOs and their originating tissue. Consistently, we observed a decrease in macrophages after a 7‐day in vitro culture (Figure 3G,H; Table S5, Supporting Information). PVOs contained lymphoid and innate immune cells through serial passage for 21 days but their expression progressively declined and did not persist beyond 30 days (Figure S3B, Supporting Information). This phenomenon may have resulted from the lack of additional interleukin stimulation in our culture medium, which is consistent with other reports.^[^ ^21^ ^]^ These findings suggest that PVOs can be a valuable tool for studying immune cells at the maternal‐fetal interface during the early stages of pregnancy, but caution should be taken when interpreting results obtained after prolonged culture periods (>21 days).

### 2.4 PVO Harbor Functional Trophoblast Lineages Which can be Differentiated Directly into EVTs

**Figure 4:**
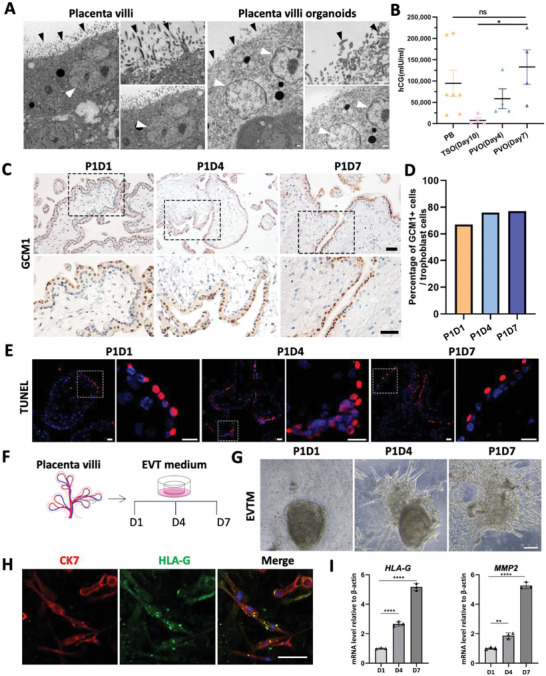
PVOs robustly secret hCGs and can be differentiated directly to EVTs. A) Electron microscopy images of the first‐trimester placenta villi and PVOs. Surface microvilli were indicated with arrowheads and multinucleated areas were indicated with white arrows. Scale bars, 1 µm. Experiments were performed in triplicates. B) Chemiluminescent immunoassay for hCG‐β detection in PVOs at days 4 and 7 after differentiation. The amount of hCG‐β produced by trophoblast organoids (between days 7 and 10 after passaging) in 72 h is shown. PB, Peripheral blood. C) Immunochemistry staining of GCM1 in the sections of PV and PVOs (Day 1, 4, and 7). Scale bar, 100 µm. D) The relative percentage of GCM1^+^ cells in trophoblast cells at P1D1, P1D4, and P1D7 PVOs. E) TUNEL staining in the cultured PVOs. Scale bars, 10 µm. F) Scheme depicting EVT differentiation in PVOs. G) Phase‐contrast images of PVOs on day 1, day 4, and day 7 after EVT differentiation. Scale bars, 100 µm. H) Immunostaining for CK7 and HLA‐G at day 7 of EVT differentiation. Scale bars, 50 µm. I) Relative expression of HLA‐G and MMP2 in PVO‐EVT using quantitative real‐time RT‐PCR. Biological replicates, *n* = 3. **p < 0.01, **** *p* < 0.0001, ns, not significant.

To assess the secretory function of PVOs, electron microscopy was used to detect STBs which were believed to secrete critical pregnancy hormones into maternal circulation, such as hCG and placental lactogen. As shown in **Figure** **4**A, a highly polarized epithelial layer covered with microvilli and multinucleated nuclei with abundant secretory organelles were observed in PVOs as that of PVs. Four PVOs derived from different gestational ages (6–9 weeks) were established and the secretory activity was measured by chemiluminescent immunoassay after 4 and 7 days of culture. PVO organoid medium demonstrated considerably high levels of hCG secretion (>10000 mIU mL^−1^), which continued to rise with extended culture time (Figure 4B). Interestingly, PVOs produced substantially more hCG than TSOs derived from the same amount of placenta villi, indicating PVOs were more functional than TSOs (Figure 4B). To investigate whether the increased secretion of HCG during culturing corresponds to the formation of a great number of STB in PVOs, we stained GCM1 in the sections of PVOs and quantified the number of positive nuclei. As revealed in Figure 4C,D, no apparent increase in the percentage of STBs at day 7 compared to day 1 in culture was observed. This suggests that the elevated HCG levels were primarily a result of PVO expansion during the culturing process, rather than an indication of increased STB formation.

In human placenta, the STB undergoes highly regulated turnover process, involving the fusion of CTBs into the STB layer and shedding of portions of the STB into the maternal circulation.^[^ ^15^, ^22^ ^]^ To assess whether this turnover occurs in our PVO system, we detected apoptosis, a normal constituent of cell turnover,^[^ ^23^ ^]^ via performing TUNEL staining on PVO sections. The result revealed that apoptosis predominantly occurred in the STB layer during in vitro culture (Figure 4E), while the inner trophoblast layer, known as CTBs, exhibited active proliferation (Figure 2B), indicating that our PVO system accurately mimics the turnover process of STB in an in vitro setting.

Previous studies have proved that EVTs can be induced from cultured trophoblast cells or organoids using EVT medium.^[^ ^9b,d^ ^]^ By adapting this EVT differentiation protocol, we cultured PVOs plated on the ALI surface with EVT medium in the lower chamber and assessed EVT morphology and markers at days 1, 4 and 7 (Figure 4F). After 4 days of EVTM culture, differentiated EVTs migrated out of the PVO surface and invaded into the Matrigel to form tracks, eventually, the EVTs adhere to the plate after 7 days in EVTM (Figure 4G). Immunofluorescence staining for HLA‐G, a marker for EVT cells,^[^ ^24^ ^]^ revealed that CK7^+^HLA‐G^+^ staining on the protrusions that migrate out of the PVOs (Figure 4H). Quantitative PCR demonstrated an apparent increase in HLA‐G and MMP2 levels, confirming efficient EVT induction upon exposure of organoids to EVTM (Figure 4I).

### 2.5 Placenta Villi Organoids Faithfully Recapitulate PV at the Genomic Level

**Figure 5:**
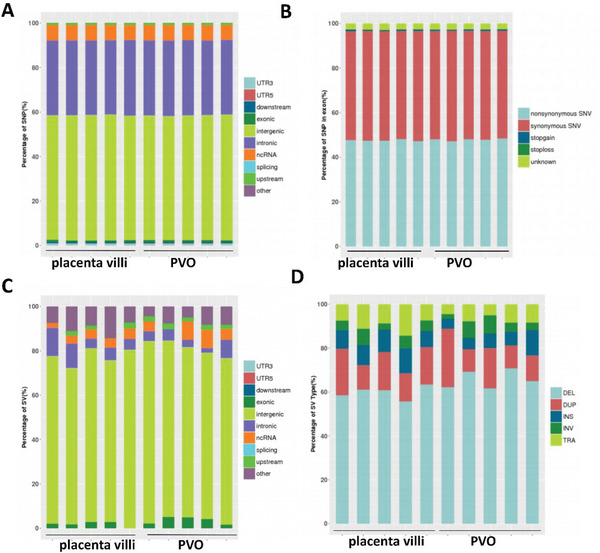
PVOs maintain genomic landscape of the corresponding placenta. A) Genome‐Wide detection of single nucleotide polymorphism (SNP) and structural variation (SV) to reveal the genomic characteristics of early placenta and PVOs. B) The proportion distribution of SNPs in each region, and the functional classification of SNPs in the exon regions of each sample. C) The distribution of SV Variations was detected in PV and PVOs in different regions of the genome. D) The percentages of different types of SVs including chromosome deletion (DEL), chromosome doubling (DUP), chromosome insertion (INS), chromosome inversion (INV), and interchromosome translocation (TRA).

The placenta is known to exhibit a high degree of mutagenesis, with multiple chromosomal aberrations affecting 1–2% of pregnancies.^[^ ^25^ ^]^ To assess whether PVOs accurately reflect the genomic landscape of the primary tissues, we performed whole‐genome sequencing (WES) on five paired PVO lines and corresponding placenta tissues. Somatic single nucleotide variants (SNVs) and structural variants (SVs) were identified and analyzed. By classifying SV variants, we found that the proportions of chromosome deletion (DEL), chromosome insertion (INS), chromosome doubling (DUP), chromosome inversion (INV), and interchromosome translocation (TRA) were similar in both tissues and PVOs (**Figure** **5**A,B). CNVs exist in both tissues and PVOs genomes with a similar distribution (Figure 5C,D). Together, PVOs maintained genomic landscape of corresponding placenta tissues. In general, most SNVs and SVs present in original placenta tissues were maintained in PVOs derived from them.

### 2.6 Preeclampsia Derived PVOs Resemble the Pathological Features

**Figure 6:**
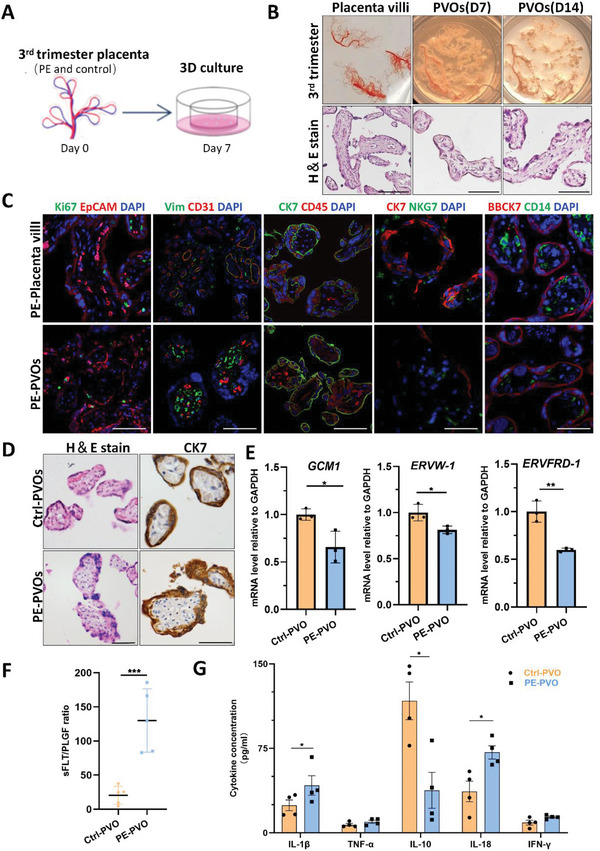
PVOs derived from placenta of preeclampsia patients resemble the pathological features. A) Scheme depicting the culture of PE/Control placenta villi organoids. B) Representative bright‐field and H&E staining images of PE placenta villi, PE‐PVOs at D7 and D14. Scale Bar, 50 µm. C) Representative confocal microscopy images of EpCAM, Ki67, Vimentin, CD31, CK7, CD45, NKG7, and CD14 in PE placenta and PE‐PVOs cultured for 7 days. Experiments were performed in six biological replicates. Scale Bar, 50 µm. D) Representative images of H&E and immunochemistry staining for CK7 in Control‐derived and PE‐derived PVOs (cultured for 7 days). Scale Bar, 50 µm. E) Relative expression of *GCM1*, *ERVW‐1* and *ERVFRD‐1* in Ctrl‐PVOs and PE‐PVOs. ** *p* < 0.01, * *p* < 0.05. F) The ratio of sFLT/PLGF levels in the supernatant of Ctrl‐PVOs and PE‐PVOs at day 7 of culture. *** *p* < 0.001. G) Relative levels of IL‐1β, IFN‐γ, TNF‐α, IL‐10, and IL‐18 in the culture medium supernatant of PE‐PVOs compared to control‐PVOs using flow multifactor detection assay. * *p* < 0.05. Experiments were performed in 4 biological replicates.

Early onset preeclampsia (EOPE) has been linked to poor placentation with endothelial dysfunction, inappropriate angiogenesis, inadequate trophoblast invasion and spiral uterine artery remodeling as key contributors.^[^ ^26^ ^]^ To evaluate the practicability of our functional PVO model, we derived PVOs from placenta villi of EOPE and age‐matched preterm birth pregnancies (**Figure** **6**A). As EOPE is usually diagnosed after 20 weeks of gestation,^[^ ^27^ ^]^ we first evaluated the stability of our PVO system in late pregnancies. Our results demonstrated that although PVs from late pregnancies had fewer proliferative cells, their cellular components were well‐preserved, and they exhibited intact villous morphology and molecular expression patterns, such as CD31, CD45, and CK7, similar to those of the source PVs (Figure 6B–D).

The importance of HERV envelope proteins, syncytins 1 and 2, in the fusion of trophoblasts and the development of the placenta has been widely recognized.^[^ ^28^ ^]^ The mRNA and protein levels of syncytins were decreased in placentas of PE patients.^[^ ^29^ ^]^ Consistently, we observed a decrease in the expression of syncytin 1 and syncytin 2 in the placenta villi of EOPE patients, this feature was also evident in PE‐derived PVOs (Figure 6E). In addition, PE is characterized by increased levels of soluble fms‐like tyrosine kinase 1 (sFlt‐1) and decreased levels of placental growth factor (PlGF) in maternal serum. The sFlt‐1 to PlGF ratio is used as a predictor of preeclampsia.^[^ ^30^ ^]^ To gain further insights, we analyzed the supernatant of PE PVO cultures and measured sFlt‐1 and PlGF levels. As shown in Figure 6F, we observed a significant increase in the sFlt‐1/PlGF ratio in PE PVOs, consistent with observations in PE patients.

The pro‐ and anti‐inflammatory cytokines play crucial roles in the development and functions of placenta.^[^ ^31^ ^]^ It is generally agreed that preeclampsia is associated with both local and systemic changes in type 1/type 2 cytokine balance compared to normal pregnancy.^[^ ^32^ ^]^ To investigate whether this model can shed light on the mechanism of preeclampsia, we detected the levels of inflammation cytokines in the conditioned medium of PVOs using a human inflammation panel that included cytokines such as IL‐1β, TNF‐α, IL‐10, IL‐18, IFN‐γ, IL‐8, IFN‐α2, MCP‐1, IL‐6, and IL‐12. As shown in Figure 6G, we observed a significant increase in the levels of IL‐1β and IL‐18 in PVOs from PE cases (PE‐PVO) compared to controls (*P* < 0.05) (Figure 6G), while the level of IL‐10 showed significant downregulation, which was consistent with previous findings.^[^ ^33^ ^]^ Moreover, the PVOs derived from the 3rd‐trimester placenta kept the ability to expand. On day 7 of passage 2, the Ctrl‐PVO and PE‐PVO maintained the villi‐like structure and retained the Ki67^+^ CTBs (Figure S4A, Supporting Information).

## 3 Discussion

Despite attempts to recapitulate the trophoblast growth and differentiation in vitro,^[^ ^9b,d^ ^]^ functional experimental models are still required to gain a deeper understanding of the human placenta. In this study, we established a time‐efficient and cost‐effective culture system for creating multicellular, functional PVOs that can be sustained for 14–30 days or more. Our approach facilitates the expansion of trophoblast stem cells and stroma components, resulting in PVOs that closely resemble the villous placenta atomically, functionally, and genetically. Moreover, we successfully maintained stromal immune cells within the PVOs, providing a more comprehensive model for studying the pathogenesis of placenta‐originated diseases such as EOPE, miscarriage and RSA. Our findings suggest that these PVOs represent a valuable tool for investigating the development and function of the human placenta.

ALI based organoids have revolutionized the generation of organoids with tissue‐specific properties and have been proven to be an efficient model for personalized medicine.^[^ ^14^, ^34^ ^]^ In our experience, we successfully completed the culture system within one hour from extraction to plating, using mechanical dissociation without enzymatic digestion. Given the preservation of an intact stroma layer, the niche factors such as Chir‐99021, Noggin, R‐spondin1,^[^ ^35^ ^]^ which are required for trophoblast stem cell or TSO proliferation and growth, were less used in our culture system, thus lowering the cost when compared to TSO or 2D TS models. Compared to the existing TSO model, our PVOs system surpasses existing placenta‐derived organoid models by faithfully replicating cellular components and genetic alterations, preserving trophoblast stemness for long‐term culture, and maintaining intrinsic stromal immune cells. These advancements enhance our ability to study and comprehend the complex cellular interactions and microenvironment at the maternal‐fetal interface.

Trophoblast stem cells in our PVOs retain their differentiation function into EVTs and STBs similar to 2D and 3D trophoblast cell lines.^[^ ^4^, ^9^ ^]^ During pregnancy, STBs produce hCG, which is typically detected at 10–11 days after conception and peaks at 10 weeks.^[^ ^36^ ^]^ In our PVOs, the level of hCG was significantly higher at day 4 than in TSOs derived from the same amount of early placenta villi, indicating their potential as a reliable model for studying trophoblast stem cell differentiation in a more in vivo‐like environment. Moreover, upon in vitro EVTM induction, cytotrophoblasts enriched in our PVOs differentiated into EVTs, further validating their usefulness as a model system. Notably, previous proliferative cytotrophoblast cell lines were successfully derived from first‐trimester placental samples, but not term placentas.^[^ ^9b^ ^]^ Therefore, our system offers an alternative for generating trophoblast stem cell lines from not only early placenta tissues, but also late‐stage or diseased placentas.

Our ALI‐based PVO differs fundamentally from placental explants in several ways.^[^ ^37^ ^]^ First, the in vitro culture of placental explant was directly put the explant on the bottom of wells which differ from the in vivo situation. Furthermore, the medium used for placenta explant culture does not support the self‐renew of trophoblast stem cells, resulting in an extensive degeneration of syncytium and cytotrophoblast within just 4–5 days of culture.^[^ ^38^ ^]^ As a result, cultured placenta explants are not suitable for long‐term culture and passaging. Although the flow system used in placenta explant culture preserves a better placenta villi structure compared to static culture, the explants still appear damaged as early as 24 h after culture.^[^ ^39^ ^]^ In contrast, our PVO system preserves the stemness and differentiation potency of trophoblast stem cells even with long‐term culture. Of particular interest, reseeding the trophoblast cells of ALI PVOs into Matrigel droplet and culturing with TOM medium would create a TSO model which can be passaged extensively. Additionally, our PVO system preserves endogenous immune cell types such as T cell, NK cell, APCs, and macrophages, without the need to reconstitute them with trophoblast cells. This is important because the placenta is an immune‐privileged site that provides a protected environment for the developing fetus, and the unique properties and functions of the immune cells present in the placenta cannot be reflected by coculturing with immune cells derived from the periphery.^[^ ^40^ ^]^

PE is a multisystemic disorder relating to the imbalance of the local immune microenvironment of the maternal‐fetal interface. While the number of changes in macrophages and NK cells during PE is still under debate,^[^ ^20^, ^41^ ^]^ the presence of an inflammatory microenvironment is commonly observed in patients with PE. Consistently, our study found that cytokine factors IL‐1β and IL‐18 were significantly elevated in the PE‐PVO group compared to the control group, providing further evidence of this inflammatory response. Additionally, we observed a significant increase in the sFlt‐1/PlGF ratio and a downregulation of syncytium markers (GCM1, ERVW‐1, and ERVFRD‐1) in PE‐PVOs, which strongly supports the representation of multiple pathogenic features associated with PE. These patient‐specific PVOs are invaluable tools for drug screening and precision medicine applications, enabling us to develop targeted treatments for disease prevention with enhanced accuracy.

Maintaining the long‐term survival of stroma cells and immune cells within multicellular organoids remains a significant challenge.^[^ ^34^, ^42^ ^]^ To overcome this hurdle, more efforts such as optimizing the medium and incorporating microfluidic organ‐on‐chip technology,^[^ ^43^ ^]^ are required to understand how to sustain the function and viability of these cells over time. Nevertheless, we have successfully developed ALI‐based PVOs from normal and diseased placentas spanning different gestation stages. Our PVO system provides a valuable tool to investigate cell–cell interactions within an immune intact environment for several weeks. Upscaling the PVO models could greatly promote the development of new diagnostic and curative treatments for pregnancy disease.

## 4 Experimental Section

### Sample Collection

All tissue samples used for this study were obtained with written informed consent from all participants. The study was approved by the Medical Ethics Committee of the Third Affiliated Hospital of Guangzhou Medical University, Medical Research (No. 2018002). First trimester placentas (6–9 weeks gestation) of healthy (normal pregnancies without a history of miscarriage) and RSA (two or more previous spontaneous unexplained abortions, normal karyotype of parents and abortus, and absence of uterine malformation, endocrine, metabolic, autoimmune diseases, or infection) women were obtained from elective termination of pregnancy. Third‐trimester placentas (28–34 weeks of gestation) were obtained after elective cesarean section. Among them, 6 cases as normal control, and 10 cases as PE samples. Human peripheral blood was collected on the day of admission. Additional sample information is available in Table S1 (Supporting Information).

### Tissue Preparations

Placenta villi (1–2 g) were macroscopically identified and washed in phosphate‐buffered saline to remove excess blood. For the 1st trimester placenta, sterile ophthalmic tweezers or surgical blades were used to scrape small branches of villi from the chorionic plate and decidua basalis. Villi were then minced finely on ice. For the 3rd trimester placenta, a whole layer of tissue measuring around the size of 1 cm × 1 cm was cut longitudinally with sterile scissors from the placenta close to the attachment of the umbilical cord. After rinsing with ice‐cold saline, the placental tissue was selected from the maternal surface and the chorionic branches were separated with ophthalmic scissors under an anatomical microscope. Samples were divided into four parts, which are processed for histopathological analysis, RNA and DNA isolation, dissociated into single cells and the remaining processed for organoid derivation.

### Placenta Villi Organoid Culture

Collagen solutions were mixed as follows: Cellmatrix I‐A (Wako, 637‐00653), Advanced DMEM/F12 (Gibco, #12634010), 20 × 10^−3^ m HEPES (Thermo Fisher Scientific, #15630080) at a ratio of 8:1:1. The mixture was kept on ice to prevent gel formation until use. The formation of air bubbles should be avoided during mixing.

To prepare the culture dish, Millicell culture plate inserts (Millipore, #R1KB36634) with permeable membrane bottoms were placed in a 60 mm tissue culture dish. To create the bottom layer, 1 mL of the prepared reconstituted collagen solution was added to the inserts under sterile conditions. The culture dish with the inserts was incubated in an incubator at 37 °C for 30 min.

Villi slices or villi were washed with 10 mL basal medium and resuspended ≈0.1 mg in 1 mL reconstituted collagen solution. The mixtures were layered on top of the presolidified bottom layer to form the double dish air–liquid culture system as described. 2 mL placenta villi organoid culture medium was added into the culture dish on the outer layer. The culture medium was changed twice a week.

The basal medium consisted of Advanced DMEM/F12 (Gibco, #12634010), 10 × 10^−3^ m HEPES (Thermo Fisher Scientific, #15630080), 1× GlutaMAX (Life Technologies, #2268102), and 1% penicillin/streptomycin (Gibco, #15140‐122). The placenta villi organoid culture medium consisted of the basal medium supplemented with 10 × 10^−3^ m nicotinamide (Sigma, #N0636), 1X B‐27 (Invitrogen, #12587010), 1 × 10^−3^ m *N*‐acetylcysteine (Sigma, #A9165), 10 × 10^−6^ m SB431542 (Wako, #031‐24291), 0.5 × 10^−3^ m A83‐01 (Wako, #039‐24111), 100 ng mL^−1^ EGF (Wako, #053‐07871), 100 ng mL^−1^ FGF2 (Peprotech, #450‐33), 50 ng mL^−1^ HGF (Peprotech, #100‐39), 2 × 10^−6^ m Y‐27632 (Wako, #030‐24021), 2.5 × 10^−6^ m PGE2 (Sigma,#P0409), and 50% (v/v) WRN‐CM. WRN‐conditioned medium was obtained from a commercially available ATCC CRL‐3276 (ATCC) cell line.

Organoids were passaged every one to two weeks. The upper and bottom layers were transferred to another 60 mm culture dish with tweezers and the organoids in collagen layers were minced with sterilized scissors and scalped on the dish for 5 min at room temperature. Organoids were dissociated with 1 mg mL^−1^ collagenase IV (Sigma, #C5138) at 37 °C for 30 min, followed by three 5 min washes with 100% FBS (BI, # 04‐001‐1ACS) and replating at a 1:2–1:3 ratio into new air–liquid interface collagen gels.

### Histology and 3D Tissue Clarity

Tissues were fixed in 4% paraformaldehyde (PFA) at 4 °C overnight. Organoids were fixed in 4% PFA for 15 min and placed in 20–50 µL Histogel (Epredia, #204235) before tissue processing and embedding. Formalin‐fixed paraffin‐embedded (FFPE) tissue sections (5 µm) were de‐paraffinized, rehydrated, and then stained with hematoxylin and eosin (H&E) for histological analysis.

3D Clarity was implemented using CytoVista Tissue Clearing Kit (Invitrogen, #V11315) according to the manufacturer's protocol. In brief, organoids were fixed in 4% PFA at room temperature for 2 h, washed twice in PBS, then once in 50% methanol diluted with PBS, 80% methanol in deionized water, 100% methanol, 20% DMSO/methanol, 80% methanol in deionized water, 50% methanol in PBS, 100% PBS, and finally in PBS with 2% Triton X‐100. Samples were blocked with 2% BSA (SolarBio, #919A053) at 37 °C and incubated with primary antibody (CK7, Abcam, #181598, 1:200 dilution) at 4 °C for 7 days with gentle shaking. After washing 6 times in PBST, samples were incubated with secondary antibody (1:500 dilution, Cell Signaling Technology, #4413) for 3 days at 4 °C with gentle shaking. Organoids were washed with 50% methanol in PBS, then 80% methanol in deionized water, and finally 100% methanol with gentle shaking. CytoVista 3D Cell Culture Clearing Reagent was added to the organoids for 5 min before imaging using opera phoenix (PerkinElmer).

### Immunostaining

FFPE tissue sections (5 µm) were deparaffinized, rehydrated, and subjected to immunohistochemistry. For antigen retrieval, slides were autoclaved in citrate buffer (pH 6.0) or EDTA (pH 9.0). Endogenous peroxidase activity was quenched in 3% H_2_O_2_ in methanol for 15 min, and sections were blocked with 5% BSA/PBS for 1 h at room temperature. The sections were incubated with primary antibodies at 4 °C overnight, washed with PBS (3 times, 10 min each), incubated with HRP‐labeled secondary antibodies for 1 h at room temperature, and washed again with PBS (3 times, 10 min each). Signals were visualized using the DAB Peroxidase (HRP) Detection Kit (ZSGB‐Bio, ZLLI‐9019). Primary antibodies were listed as below: CK7 (1:500, #ab181598, Abcam), CDH1 (1:200, #3195S, Cell Signaling Technology), Vimentin (1:200, #5741S, CST), TFAP2α (1:200, #ab108311, Abcam), CGB (1:200, #AP13036B, Abcepta), Ki67 (1:200, #ab15580, Abcam), GCM1 (#HPA011343, Sigma, 1:100). Secondary antibodies including goat antimouse IgG‐HRP (1:500, Cell Signaling Technology, #7076) and goat antirabbit IgG‐HRP (1:500, Cell Signaling Technology, #7074) were used. For immunofluorescence, sections were incubated with primary antibodies at 4 °C overnight, washed in PBS (3 times, 10 min each), and followed by incubation with DAPI (2 µg mL^−1^) and secondary antibodies. After washing, samples were mounted with ProLong Gold Antifade reagent (Life Technology, #P36930). Primary antibodies including CK7 (#ab192077, Abcam, 1:300), TFAP2C (#SC12762, Santa Cruz, 1:100), CD31 (#3528S, Cell Signaling Technology, 1:200), GATA3 (#5852S, Cell Signaling Technology, 1:200), CD45 (#250015, ZENBio, 1:100), CD14 (#ab181470, Abcam, 1:200), CD86 (#ab220188, Abcam, 1:200), CD163 (#ab182422, Abcam, 1:200), CD3 (#ab16669, Abcam, 1:200), HLA‐G (#GTX78335, Gentex, 1:100) were used. Secondary antibodies were used, including goat antimouse IgG, Alexa Fluor 647 conjugate (A28181, Thermo Fisher Scientific, 1:500), goat anti‐rabbit IgG, Alexa Fluor 555 conjugate (A27039, Thermo Fisher Scientific, 1:500). Confocal fluorescence images were captured using a Nikon A1R laser scanning confocal Ti2 microscope.

### Flow Cytometry

Placenta single cells were obtained as described above. PVOs were dissociated with 200 units mL^−1^ collagenase IV (Sigma, #C5138) at 37 °C for 30 min, centrifuged at 300 × *g* for 5 min, and cell pellets were resuspended with 0.05% trypsin/EDTA (Gibco, #25300120). After digestion for 10 min, cells were collected by centrifuge and washed twice with PBS. Samples were stained with 1 µL BD Horizon Fixable Viability Stain 700 (BD Biosciences, #564997) and incubated for 20 min at 4 °C, then washed with 2% FBS/PBS and stained with 5 µL FcR blocking reagent (BD Biosciences, #564219) for 20 min at 4 °C. Antibodies were added to the cell suspension and incubated for 45 min at 4 °C. Meanwhile, the UltraComp eBeads (Thermofisher, #01‐2222‐42) were vortexed for at least 30 seconds, and one drop of beads was dispensed into an Eppendorf tube. Various antibodies in the Table S5 (Supporting Information) were added as the manufacturer recommended, followed by cell spin‐down and resuspension with FACS buffer. Flow cytometry was carried out using Beckman CytoFlex, data were analyzed using FlowJo software (version 10.8) and all compensation was applied digitally after acquisition. The gating strategy and antibodies used are listed in Figure S2 and Table S2 (Supporting Information).

### Differentiation of PVOs

For the induction of EVT cells, PVOs were cultured in 2 mL PVOs culture medium. On day 1, the medium was replaced with the EVT medium. EVT medium consisted of DMEM/F12 (Gibco, #12400‐024) supplemented with 0.3% BSA (Sigma, #A9418), 0.1 × 10^−3^ m 2‐mercaptoethanol (Gibco, #21985023), 100 ng mL^−1^ NRG1 (RD, #5898‐NR), 7.5 × 10^−3^ m A83‐01 (Wako, #039‐24111), 2.5 × 10^−3^ m Y27632 (Wako, #030‐24021), 0.5% Penicillin–Streptomycin (Gibco, #15140‐122), 1% ITS‐X supplement (Gibco, #2034570), and 4% Knock Out Serum Replacement (Gibco, #A3181502). On day 4 and 7, EVT‐PVOs were collected for analysis.

### RNA Isolation and Reverse Transcription Polymerase Chain Reaction (RT‐PCR)

Total RNA was extracted using TRIzol (Takara, #T9108) according to the manufacturer's protocol. Extracted RNA was diluted with DEPC‐treated water and quantified using a ratio of measurements at 260 and 280 nm (Nanodrop). Then RNA was reversed to cDNA using a PrimeScript RT reagent Kit with gDNA Eraser (Takara, #RR047A) according to the manufacturer's instructions. TB Green Premix Ex Taq (Tli RNaseH Plus) (Takara, #RR420A) was used for quantification, with primer sequences provided as following. All real‐time PCR (qPCR) reactions were carried out using the Q3 Real‐Time System (Applied BioSystems). The results were normalized to the expression of GAPDH. Relative fold changes in gene expression were calculated using the 2−ΔΔCt method, normalized with respective controls. Primers for qRT‐PCR analysis of EVT‐PVOs were shown as following:

Human GAPDH (F: ACATCGCTCAGACACCATG R: TGTAGTTGAGGTCAATGAAGGG)
Human HLA‐G (F: CCACCACCCTGTCTTTGACTAT R: ACGTCCTGGGTCTGGTCCT)
Human MMP2 (F: TGGCACCCATTTACACCTACAC R: ATGTCAGGAGAGGCCCCATAGA)
Human CGB (F: CAGCATCCTATCACCTCCTGGT R: CTGGAACATCTCCATCCTTGGT)
Human SDC1 (F: CTATTCCCACGTCTCCAGAACC R: GGACTACAGCCTCTCCCTCCTT)
Human GCM1(F: TTCTCCAAGAGTTATGGTCTGGG R: CCACGCTTGTAGATCGCCA)
Human ERVW‐1 (F: CCAAATTCCCTCCTCTCCTC R: CGGGTGTTAGTTTGCTTGGT)
Human ERVFRD‐1(F: CTACCCCAACTGCGGTTAAA R: GGTTCCTTTGGCAGTATCCA)

### Chemiluminescence Immunoassay

For the first trimester placenta, placenta villi were extracted from patients who underwent elective termination of normal pregnancies at 6 weeks, 7 weeks, 8 weeks, and 9 weeks. Medium supernatants of PVOs were collected after being cultured for 4 days and 7 days, while those of trophoblast organoids were collected at 10 days after passage. Patients’ serum was collected on the day of surgery as control. The amount of secreted hCG was measured using a chemiluminescence immunoassay (#A015,DARUI biotechnology).

For the third trimester and PE placenta, the placenta was obtained from women undergoing elective cesarean section and cultured as described previously. Seven days after culture, the medium supernatants of Ctrl‐PVO and PE‐PVO were collected, and the levels of sFLT (# A183, DARUI biotechnology) and PLGF(# A183, DARUI biotechnology) were identified by chemiluminescence immunoassay.

### TUNEL Detection

The TUNEL assay was conducted using the TUNEL detection kit (#A113, NOVIZAN Biotechnology) according to the instructions. Briefly, paraffin‐embedded tissue sections were routinely deparaffinized, hydrated, and incubated with 20 µg mL^−1^ Proteinase K at room temperature for 20 min, then washed three times in PBS for 5 min per wash. 1 × Equilibration Buffer was added to the section, and equilibrated at room temperature for 30 min. After removing Equilibration Buffer, the TdT reaction mixture was dropped on the sample, and incubated at 37 °C for 60 min. The slides were washed in PBS 3 times, 5 min each. Slides were counterstained in the dark with 2 µg mL^−1^ DAPI for 10 min at RT. The pictures were captured by Nikon confocal microscope.

### Electron Microscopy

Samples from human placentas were fixed by immersion in 2.5% glutaraldehyde overnight. Placenta villi organoids (at day 7) were fixed directly with 0.5% glutaraldehyde, 0.2 m sodium cacodylate buffer (pH 7.2) for 30 min. All samples were divided into long strips with a cross‐section of 1 mm^2^ and a length not exceeding 3 mm. Secondary fixation was achieved by immersion in 1% osmium tetroxide in 1,4‐piperazinediethanesulfonic acid (PIPES)buffer for 1 h at room temperature. After washing, the specimens were dehydrated in graded ethanol and embedded in Araldite epoxy resin. Ultrathin sections (100 nm) were cut on a Leica UC7. Sections were counter‐stained with uranylacetate, followed by lead citrate, before imaging in a Tecnai G2 Spirit electron microscope (FEI).

### Detection of Multi‐Inflammatory Cytokines

Detection of inflammatory cytokines was performed using the Multi‐Analyte Flow Assay Kit (human inflammation panel 1, Biolegend, #740808) following the manufacturer's instructions. In brief, medium supernatants of PE‐PVOs and Control‐PVOs were collected after culturing for 7 days, then diluted 1:1 with assay buffer. 25 µL mixed beads were added to wells and incubated for 2 h at room temperature with 800 rpm shaking. The wells were washed twice with 1× Wash Buffer, followed by detection antibodies incubation and SA‐PE incubation. The beads were washed twice and resuspended with 150 µL of 1× Wash Buffer. Samples were read on a Attune NxT Flow cytometer (Invitrogen).

### DNA Isolation and Whole Genome Sequencing

The total DNA of tissues or organoid cultures (at day 7) was extracted using DNeasy Blood and Tissue Kit (Magentec, #D3125) according to the manufacturer's protocol. Illumina DNA PCR‐Free Library Preparation kit was used to construct the indexed paired‐end libraries and libraries were sequenced on Illumina NovaSeq 6000. SNP and INDEL were detected using the method of population site variation detection with the software GATK.^[^ ^44^ ^]^ Filtering and screening were further conducted according to quality value, depth, repeatability and other factors. DELLY^[^ ^45^ ^]^ was used for SV detection, and Control‐FREEC was used for CNV detection. The mutation sites with high confidence were finally obtained. The software ANNOVAR^[^ ^46^ ^]^ and the existing genome annotation files (gff/gtf) were used to conduct corresponding annotation for the detected mutation sites.

### 4.1 Genomics Single‐Cell RNA‐Seq

Placenta tissues were rinsed with PBS to remove obvious blood clots, cut into small pieces, and digested with 1 mg mL^−1^ collagenase IV (Sigma, #C5138), 3 mmol L^−1^ CaCl2, 10 mg mL^−1^ DNase I (Sigma, #DN25) for 30 min. PVOs were enzymatically dissociated as described above. The released cells were filtered through 70 and 40 µm mesh, centrifuged, and resuspended in 5 mL of red blood cell lysis buffer (Booster, #AR1118) for 10 min to exclude any remaining red blood cells. Finally, the pelleted cells were resuspended in PBS and used for single‐cell library preparation following 10× Genomics Chromium Single‐Cell Kits protocol.

Single‐cell libraries were sequenced on Illumina HiSeq X Ten instruments with paired‐ended 150 bp reads. Reads were processed using the Cell Ranger 4.0.0 pipeline with default parameters. FASTQs generated from the Illumina sequencing output were aligned to the human reference genome (GRCh38) using the STAR algorithm.^[^ ^47^ ^]^ Gene‐Barcode matrices were generated for each sample by counting unique molecular identifiers (UMIs) and noncell associated barcodes were filtered. Finally, the gene barcode matrix containing barcode cells and gene expression counts was generated. This output was then imported into the Seurat v4^[^ ^48^ ^]^ R toolkit for quality control and downstream analysis. All functions were run with default parameters unless otherwise specified. All mitochondrial‐encoded genes were deleted and low‐quality cells (Genes detected <250 and UMI counts <500) were excluded. 20 826 cells (10 362 for PV and 10 464 for PVO datasets) were kept for analysis. SCTransform^[^ ^49^ ^]^ were used to normalize the filtered gene‐barcode matrix . All samples were integrated with the function IntegrateData in Seurat package to account for technical and individual sample variations.^[^ ^50^ ^]^

Standard deviation saturation plot was used to determine the optimal number of principal components from the integrated dataset. The Louvain community detection‐based method was performed on integrated expression values on the shared‐nearest‐neighbor (SNN) graph as implemented in the FindCluster function of the Seurat package for clustering single‐cell data. The resolution parameter to find the resulting number of clusters was tuned so that it produced clusters large enough to capture most of the biological variability. UMAP analysis was performed using the RunUMAP function with default parameters.^[^ ^51^ ^]^ To validate whether our culture system retained immune components, 3359 cells positive were analyzed for CD45, and use t‐SNE to analyze immune cell subpopulation. Cluster‐specific gene markers were identified using Seurat's FindAllMarkers with cutoffs avg_log2FC > 0.25. Clusters were annotated using canonical cell‐type markers.

### Statistical Analysis

Where applicable, statistical methods are outlined in the respective figure legends. Statistical analysis was performed utilizing Microsoft Excel, GraphPad, and R package. Statistical significance was set at *P* < 0.05. *P* values were calculated using a two‐tailed Student's t‐test.

In the case of representative results, the number of independent organoid lines or experimental repetitions and their relevant description are indicated in the figure legend.

## Conflict of Interest

The authors declare no conflict of interest.

## Supporting information

Supporting Information

## Data Availability

The data that support the findings of this study are available on request from the corresponding author. The data are not publicly available due to privacy or ethical restrictions.
