# Supplementary material for: Generating Functional Multicellular Organoids from Human Placenta Villi
Source: Adv Sci (Weinh). 2023 Jul 12;10(26):2301565. doi: 10.1002/advs.202301565 (PMC10502861; doi:10.1002/advs.202301565)
Supplement: Supplementary file 1 — Supporting Information [file ADVS-10-2301565-s001.pdf]

ADVANCED SCIENCE
Open Access

Supporting Information

for *Adv. Sci.*, DOI 10.1002/advs.202301565

Generating Functional Multicellular Organoids from Human Placenta Villi

Lijun Huang, Zhaowei Tu, Liudan Wei, Wei Sun, Yifan Wang, Shilei Bi, Fang He, Lili Du, Jingsi Chen, Julia Kzhyshkowska, Haibin Wang, Dunjin Chen and Shuang Zhang**

Supporting information

Generating Functional Multicellular Organoids from Human Placenta Villi

Lijun Huang[$], Zhaowei Tu[$], Liudan Wei, Wei Sun, Yifan Wang, Shilei Bi, Fang He, Lili Du, Jingsi Chen, Julia Kzhyshkowska, Haibin Wang, Dunjin Chen[*], Shuang Zhang[*]

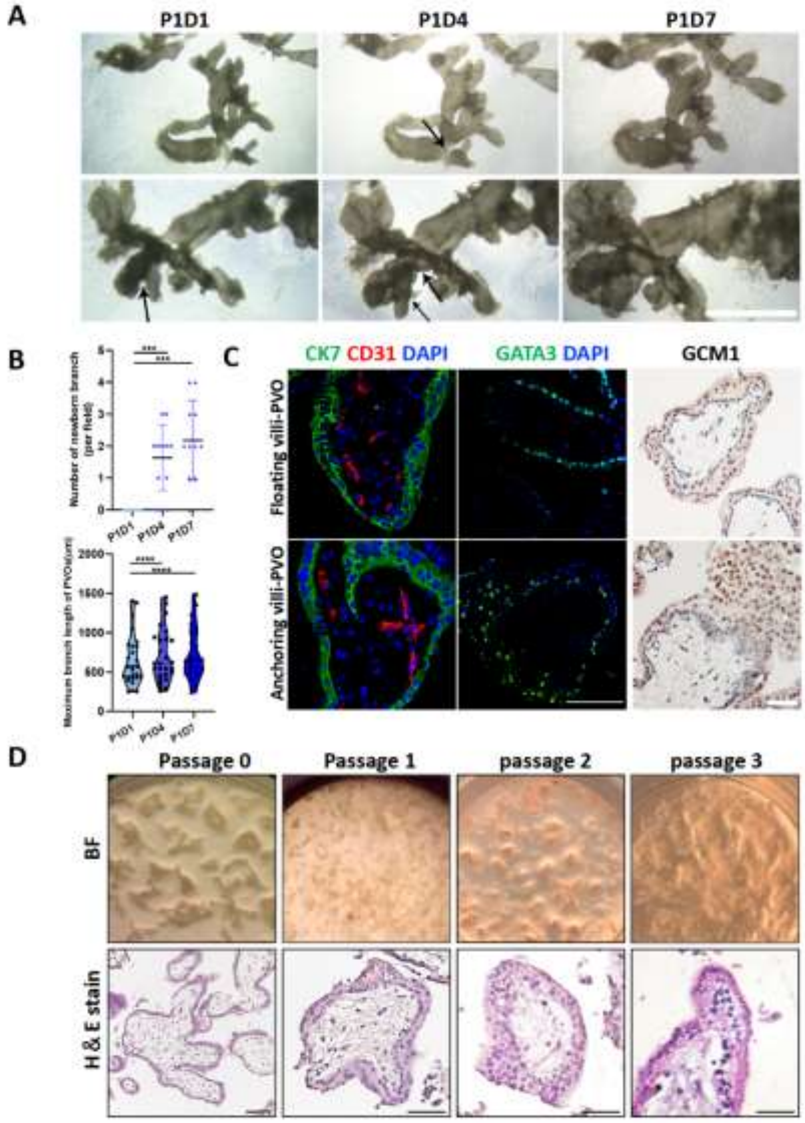

Supplementary Figure 1, Related to Figure 1

Preservation of cell components in PVOs, and long-term culture of PVO. (A) Representative pictures of growing PVOs. Scale bar, 500 µm. The arrows indicated new villi branches. (B) Quantification of the number of new branches and max branch length. ***p<0.0001***p<0.001, **p<0.01. (C) Immunostaining for CK7, CD31, GATA3, and GCM1 in the d7 Floating villi-PVOs and Anchoring villi-PVOs. CK7+ cells lined at the outer layer of the organoid and CD31+ cells were preserved in the stroma. PVOs retain the CTB (marked by GATA3) and STB (marked by GCM1). Scale bars, 100 µm. Representative images from 3 Floating villi and 6 Anchoring villi-derived organoids. (D) Bright-field images of collagen gels after seeding villi slices starting from passage 0 (day 0) to passage 3 (day 28). Scale bars, 100 µm.

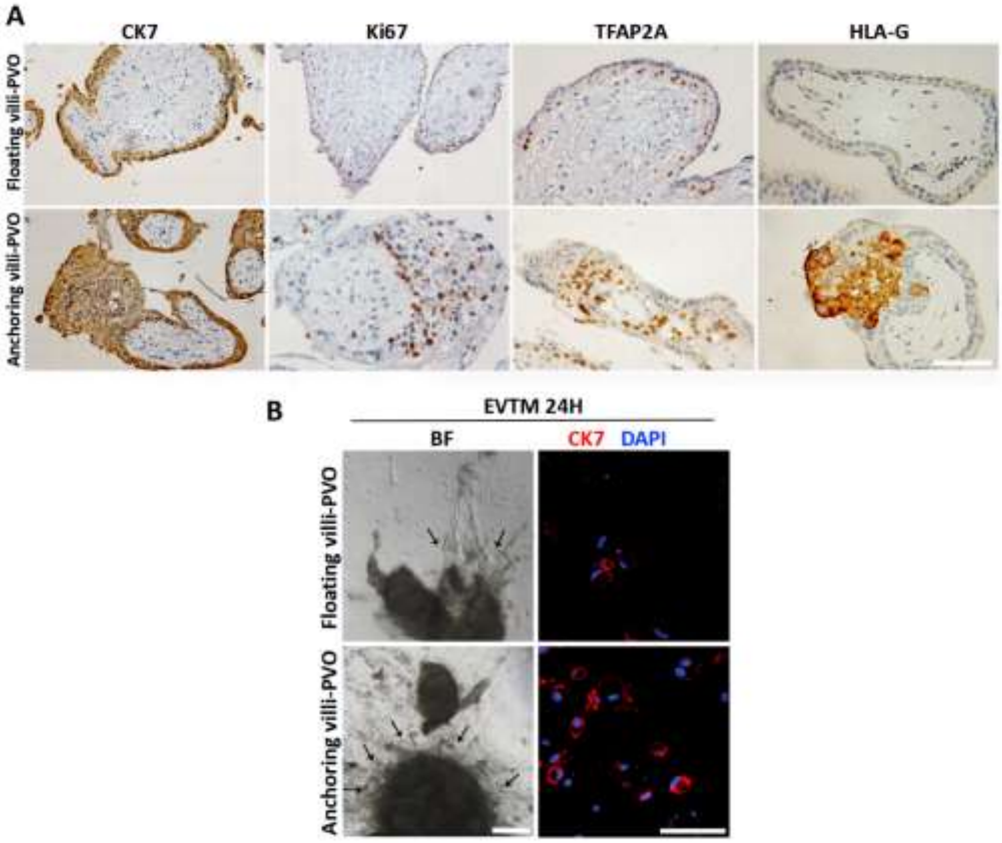

Supplementary Figure 2, Related to Figure 1

Comparisons between PVOs derived from floating villi and anchoring villi. (A) Immunohistochemistry staining for CK7, Ki67, TFAP2A and HLA-G of floating villi-PVO and anchoring villi-PVO. Representative images from n =10 (floating villi-PVO) and n =6 (anchoring villi-PVO). CTB, STB and CCC cells stain positively for CK7. Ki67 is present in the inner CTB layer in PVO and the CCC of anchoring villi-PVO. CTBs displayed nuclei-localized staining of TFAP2A, and HLA-G showed specific localization in proximal CCCs of anchoring villi. Scale bars, 50 µm. (B) Bright-field views and CK7 immunostaining pictures of floating villi PVOs and anchoring villi-PVOs under EVT differentiation medium (24H). EVT cells sprouted out from PVOs was indicated by arrows in BF images. Scale bars, 50 µm.

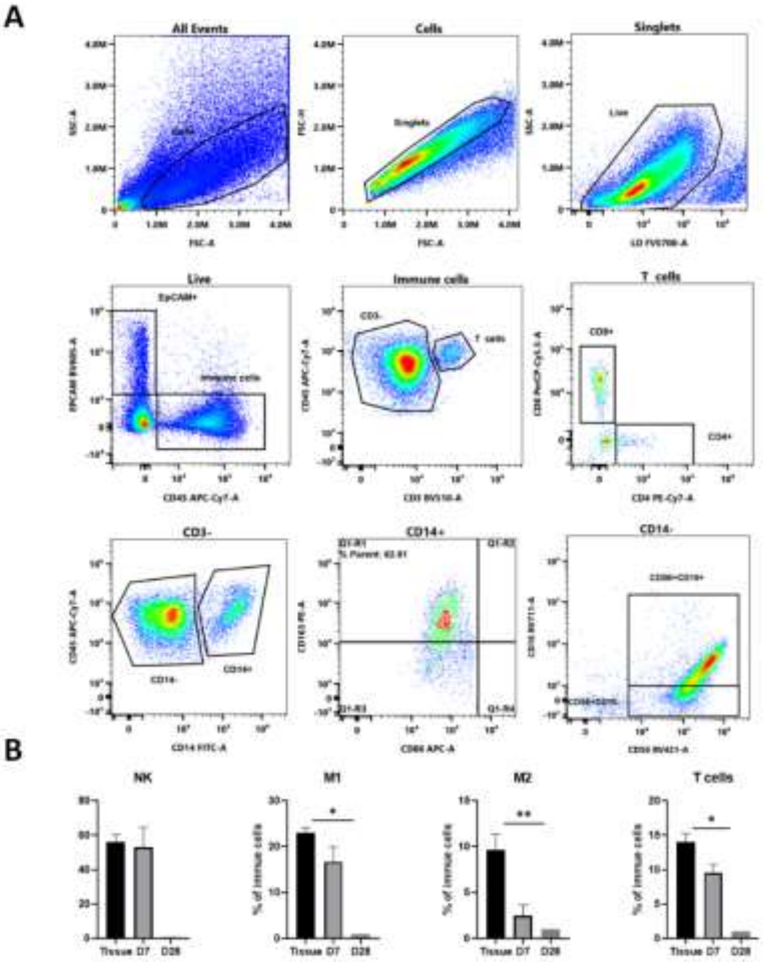

Supplementary Figure 3, Related to Figure 3

Flow cytometry profiling analysis of immune cells in PV and PVO. (A) Representative flow cytometry plots and gating strategy for immune cell analysis using a panel of 10 antibodies including CD45, CD3, CD4, CD8, CD14, CD163, CD86, CD56, CD16, Fixable Viability Stain 700 in placenta villi and PVOs. (B) the proportion of immune cells in placenta villi and PVOs based on flow cytometry analysis. The data were shown as means ± SEM. * $P < 0.05$. Experiments were performed in triplicates.

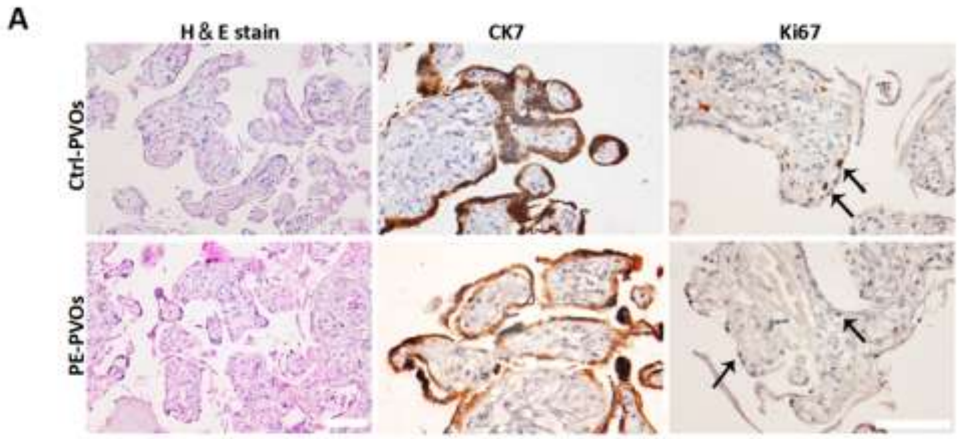

Supplementary Figure 4, Related to Figure 6

The PVOs derived from the 3rd-trimester placenta retained the capability of passage. (A) H&E, CK7, Ki67, and CD163 staining in Ctrl-PVOs and PE-PVOs derived from 3rd-trimester placenta at day 7 of passage 2. The arrows indicated the proliferating CTBs which were Ki67+. Scale bars, 100 µm.

Product	Company	Product Number	Final Concentration	Volumn
	ADMEM/F12+++			To 10ml
	WRN conditioned media			5 ml
Nicotinamide	Sigma	N0636	409.4mM	244.3 ul
N-Acetylcysteine	Sigma	A9165-5G	612.8mM	16.3 ul
B-27 without vitamin A	Life Technologies	12587010	50X	200 ul
A83-01	wako	039-24111	5mM	1 ml
SB431542	Wako	031-24291	5 mM	20 ul
EGF	wako	053-07871	0.1mg/ml	10 ul
FGF2	Peprotech	450-33-50ug	0.1mg/ml	20 ul
HGF	Peprotech	100-39	50ug/ml	10 ul
Y-27632	Wako	030-24021	50mM	0.4 ul
PGE2	Sigma	P0409	25mM	1 ul

Table S1, Culture Conditions for Placenta villi organoids (PVO), Related to Figure 1

Patient	Group	Age	Resource	Gestational age	Dignosis
1	First trimester (control)	26	selective abortion	8	Intrauterine pregnancy， live birth
2	First trimester (control)	21	selective abortion	7	Intrauterine pregnancy， live birth
3	First trimester (control)	32	selective abortion	9	Intrauterine pregnancy， live birth
4	First trimester (control)	26	selective abortion	6	Intrauterine pregnancy， live birth
5	First trimester (control)	24	selective abortion	7	Intrauterine pregnancy， live birth
6	First trimester (control)	40	selective abortion	8	Intrauterine pregnancy， live birth
7	First trimester (control)	35	selective abortion	6	Intrauterine pregnancy， live birth
8	First trimester (control)	25	selective abortion	7	Intrauterine pregnancy， live birth
9	First trimester (control)	22	selective abortion	6	Intrauterine pregnancy， live birth
10	First trimester (control)	30	selective abortion	7	Intrauterine pregnancy， live birth
11	First trimester (control)	26	selective abortion	7	Intrauterine pregnancy， live birth
12	First trimester (control)	33	selective abortion	8	Intrauterine pregnancy， live birth
13	First trimester (control)	35	selective abortion	7	Intrauterine pregnancy， live birth
14	First trimester (control)	24	selective abortion	6	Intrauterine pregnancy， live birth
15	First trimester (control)	25	selective abortion	6	Intrauterine pregnancy， live birth
16	First trimester (control)	21	selective abortion	8	Intrauterine pregnancy， live birth
17	First trimester (control)	35	selective abortion	8	Intrauterine pregnancy， live birth
18	First trimester (control)	22	selective abortion	8	Intrauterine pregnancy， live birth
19	First trimester (control)	31	selective abortion	6	Intrauterine pregnancy， live birth
20	First trimester (control)	25	selective abortion	7	Intrauterine pregnancy， live birth
21	First trimester (control)	33	selective abortion	6	Intrauterine pregnancy， live birth
22	First trimester (control)	36	selective abortion	6	Intrauterine pregnancy， live birth
23	First trimester (control)	24	selective abortion	7	Intrauterine pregnancy， live birth
24	First trimester (control)	28	selective abortion	8	Intrauterine pregnancy， live birth
25	First trimester (control)	38	selective abortion	6	Intrauterine pregnancy， live birth
26	First trimester (control)	34	selective abortion	8	Intrauterine pregnancy， live birth
27	First trimester (control)	35	selective abortion	6	Intrauterine pregnancy， live birth
28	First trimester (control)	36	selective abortion	7	Intrauterine pregnancy， live birth
29	First trimester (control)	35	selective abortion	7	Intrauterine pregnancy， live birth
30	First trimester (control)	25	selective abortion	8	Intrauterine pregnancy， live birth
31	First trimester (control)	25	selective abortion	7	Intrauterine pregnancy， live birth
32	First trimester (control)	26	selective abortion	8	Intrauterine pregnancy， live birth
33	First trimester (control)	32	selective abortion	8	Intrauterine pregnancy， live birth
34	First trimester (control)	29	selective abortion	6	Intrauterine pregnancy， live birth
35	First trimester (control)	25	selective abortion	6	Intrauterine pregnancy， live birth
36	First trimester (control)	30	selective abortion	6	Intrauterine pregnancy， live birth
37	First trimester (control)	27	selective abortion	6	Intrauterine pregnancy， live birth
38	First trimester (control)	32	selective abortion	6	Intrauterine pregnancy， live birth

39	First trimester (control)	31	selective abortion	7	Intrauterine pregnancy， live birth
40	First trimester (control)	34	selective abortion	6	Intrauterine pregnancy， live birth
41	First trimester(abortion)	35	curettage of uterine cavity	7	missing abortion
42	First trimester(abortion)	33	curettage of uterine cavity	7	missing abortion
43	First trimester(abortion)	32	curettage of uterine cavity	6	Recurrent Spontaneous Abortion
44	First trimester(abortion)	43	curettage of uterine cavity	7	Recurrent Spontaneous Abortion
45	First trimester(abortion)	34	curettage of uterine cavity	6	Recurrent Spontaneous Abortion
46	First trimester(abortion)	35	curettage of uterine cavity	6	Recurrent Spontaneous Abortion
47	First trimester(abortion)	38	curettage of uterine cavity	7	Recurrent Spontaneous Abortion
48	First trimester(abortion)	34	curettage of uterine cavity	6	Recurrent Spontaneous Abortion
49	Third trimester(Control)	31	Cesarean section	32	Preterm birth
50	Third trimester(Control)	35	Cesarean section	28	Preterm birth
51	Third trimester(Control)	27	Cesarean section	33	Preterm birth
52	Third trimester(Control)	30	Cesarean section	36	Preterm birth
53	Third trimester(Control)	25	Cesarean section	33	PE
54	Third trimester(Control)	29	Cesarean section	32	PE
55	Third trimester(PE)	37	Cesarean section	28	PE
56	Third trimester(PE)	36	Cesarean section	34	PE
57	Third trimester(PE)	36	Cesarean section	34	PE
58	Third trimester(PE)	31	Cesarean section	32	PE
59	Third trimester(PE)	37	Cesarean section	28	PE
60	Third trimester(PE)	22	Cesarean section	34	PE
61	Third trimester(PE)	30	Cesarean section	28	PE
62	Third trimester(PE)	29	Cesarean section	29	PE
63	Third trimester(PE)	29	Cesarean section	29	PE
64	Third trimester(PE)	22	Cesarean section	32	PE

Table S2. Sample information for PVOs Derivation, Related to Table 1

	p_val	avg_log2FC	pct.1	pct.2	p_val_adj	cluster	gene	avg_fc
1	0.0000	0.967561446	0.602	0.183	0.0000	VCT	PEG10	0.967561446
2	0.0000	0.825933876	0.793	0.441	0.0000	VCT	KRT18	0.825933876
3	0.0000	0.791868805	0.716	0.315	0.0000	VCT	KRT8	0.791868805
4	0.0000	0.781715933	0.554	0.176	0.0000	VCT	DSP	0.781715933
5	0.0000	0.730900586	0.617	0.213	0.0000	VCT	KRT7	0.730900586
6	0.0000	0.621601743	0.493	0.115	0.0000	VCT	GATA3	0.621601743
7	0.0000	0.606430118	0.443	0.109	0.0000	VCT	CLDN4	0.606430118
8	0.0000	0.601007481	0.451	0.097	0.0000	VCT	XAGE2	0.601007481
9	0.0000	0.582776969	0.395	0.085	0.0000	VCT	XAGE3	0.582776969
10	0.0000	0.582430773	0.395	0.051	0.0000	VCT	TENM3	0.582430773
11	0.0000	0.572987122	0.457	0.118	0.0000	VCT	MORC4	0.572987122
12	0.0000	0.56315685	0.391	0.09	0.0000	VCT	RPS4Y1	0.56315685
13	0.0000	0.560950595	0.385	0.057	0.0000	VCT	SPINT1	0.560950595
14	0.0000	0.557713035	0.375	0.06	0.0000	VCT	ITGB4	0.557713035
15	0.0000	0.552062177	0.407	0.083	0.0000	VCT	CDH1	0.552062177
16	0.0000	0.54745194	0.379	0.058	0.0000	VCT	SEMA6A	0.54745194
17	0.0000	0.529159546	0.434	0.111	0.0000	VCT	WEE1	0.529159546
18	0.0000	0.522991223	0.404	0.057	0.0000	VCT	TFAP2A	0.522991223
19	0.0000	0.514117626	0.438	0.094	0.0000	VCT	PHGDH	0.514117626
20	0.0000	0.488190839	0.369	0.051	0.0000	VCT	ANXA3	0.488190839
21	0.0000	0.486858378	0.297	0.021	0.0000	VCT	MIR205HG	0.486858378
22	0.0000	0.483790524	0.378	0.057	0.0000	VCT	ERVW-1	0.483790524
23	0.0000	0.471295643	0.38	0.076	0.0000	VCT	DSC2	0.471295643
24	0.0000	0.462575858	0.4	0.088	0.0000	VCT	RBM47	0.462575858
25	0.0000	0.459488241	0.262	0.011	0.0000	VCT	LRP2	0.459488241
26	0.0000	0.454807798	0.335	0.061	0.0000	VCT	MBNL3	0.454807798
27	0.0000	0.445845839	0.303	0.035	0.0000	VCT	VGLL1	0.445845839
28	0.0000	0.431831402	0.36	0.056	0.0000	VCT	IGF2BP3	0.431831402
29	0.0000	0.426973145	0.346	0.05	0.0000	VCT	LIN28B	0.426973145
30	0.0000	0.422470887	0.292	0.031	0.0000	VCT	DIAPH3	0.422470887
31	0.0000	0.419851338	0.331	0.045	0.0000	VCT	NLRP2	0.419851338
32	0.0000	0.417745459	0.318	0.029	0.0000	VCT	TFCP2L1	0.417745459
33	0.0000	0.415137059	0.254	0.035	0.0000	VCT	CMYA5	0.415137059
34	0.0000	0.411413038	0.277	0.023	0.0000	VCT	LINC01949	0.411413038
35	0.0000	0.400767688	0.327	0.049	0.0000	VCT	ERVH48-1	0.400767688
36	0.0000	0.397093924	0.33	0.051	0.0000	VCT	PKP2	0.397093924
37	0.0000	0.394847264	0.331	0.06	0.0000	VCT	KLF5	0.394847264
38	0.0000	0.389779776	0.288	0.045	0.0000	VCT	FOLR1	0.389779776
39	0.0000	0.367261608	0.31	0.046	0.0000	VCT	SH2D4A	0.367261608
40	0.0000	0.355055574	0.199	0.016	0.0000	VCT	DUSP9	0.355055574
41	0.0000	0.352544504	0.292	0.051	0.0000	VCT	ELF3	0.352544504
42	0.0000	0.469244673	0.361	0.081	0.0000	VCT	DSG2	0.469244673

43	0.0000	0.387811146	0.322	0.063	0.0000	VCT	ITIH5	0.387811146
44	0.0000	0.358954786	0.235	0.035	0.0000	VCT	ASPM	0.358954786
45	0.0000	0.392870571	0.271	0.049	0.0000	VCT	CSF3R	0.392870571
46	0.0000	0.418486977	0.371	0.093	0.0000	VCT	SHTN1	0.418486977
47	0.0000	0.378536377	0.345	0.078	0.0000	VCT	TSTD1	0.378536377
48	0.0000	0.373607718	0.276	0.053	0.0000	VCT	ENC1	0.373607718
49	0.0000	0.529693183	0.419	0.122	0.0000	VCT	SPINT2	0.529693183
50	0.0000	0.47537028	0.396	0.113	0.0000	VCT	CLDN7	0.47537028
51	0.0000	0.419423177	0.339	0.088	0.0000	VCT	ISYNA1	0.419423177
52	0.0000	0.539453361	0.572	0.227	0.0000	VCT	EZR	0.539453361
53	0.0000	0.560245712	0.4	0.128	0.0000	VCT	PEG3	0.560245712
54	0.0000	0.380132796	0.308	0.074	0.0000	VCT	HELLS	0.380132796
55	0.0000	0.385494156	0.379	0.105	0.0000	VCT	JUP	0.385494156
56	0.0000	0.353734201	0.28	0.064	0.0000	VCT	KRT23	0.353734201
57	0.0000	0.373678087	0.33	0.085	0.0000	VCT	NEDD9	0.373678087
58	0.0000	0.409417273	0.428	0.148	0.0000	VCT	ADK	0.409417273
59	0.0000	0.566024807	0.713	0.481	0.0000	VCT	TKT	0.566024807
60	0.0000	0.391541194	0.346	0.105	0.0000	VCT	LAMA5	0.391541194
61	0.0000	0.553396925	0.606	0.3	0.0000	VCT	S100A10	0.553396925
62	0.0000	0.595669018	0.59	0.279	0.0000	VCT	S100P	0.595669018
63	0.0000	0.390165892	0.258	0.062	0.0000	VCT	TOP2A	0.390165892
64	0.0000	0.508317761	0.481	0.204	0.0000	VCT	RASA1	0.508317761
65	0.0000	0.507928597	0.611	0.301	0.0000	VCT	CD24	0.507928597
66	0.0000	0.579438166	0.614	0.334	0.0000	VCT	SYNE2	0.579438166
67	0.0000	0.415101136	0.404	0.158	0.0000	VCT	GULP1	0.415101136
68	0.0000	0.455138338	0.494	0.211	0.0000	VCT	SLC38A1	0.455138338
69	0.0000	0.350322487	0.847	0.733	0.0000	VCT	NPM1	0.350322487
70	0.0000	0.523174924	0.607	0.318	0.0000	VCT	KRT19	0.523174924
71	0.0000	0.362823766	0.392	0.141	0.0000	VCT	RAB11FIP1	0.362823766
72	0.0000	0.383227432	0.401	0.161	0.0000	VCT	SH3YL1	0.383227432
73	0.0000	0.364952737	0.358	0.134	0.0000	VCT	CKS1B	0.364952737
74	0.0000	0.663001682	0.361	0.14	0.0000	VCT	PAGE4	0.663001682
75	0.0000	0.467617844	0.453	0.201	0.0000	VCT	PTPRF	0.467617844
76	0.0000	0.506098437	0.566	0.295	0.0000	VCT	SNHG29	0.506098437
77	0.0000	0.385879144	0.421	0.168	0.0000	VCT	PERP	0.385879144
78	0.0000	0.452401615	0.565	0.312	0.0000	VCT	AKR1B1	0.452401615
79	0.0000	0.385626231	0.433	0.199	0.0000	VCT	ARID3A	0.385626231
80	0.0000	0.362027606	0.389	0.171	0.0000	VCT	GGCT	0.362027606
81	0.0000	0.4688474	0.423	0.192	0.0000	VCT	PHLDA2	0.4688474
82	0.0000	0.406405146	0.488	0.268	0.0000	VCT	SMS	0.406405146
83	0.0000	0.357657209	0.397	0.164	0.0000	VCT	AC243919.1	0.357657209
84	0.0000	0.377166136	0.455	0.231	0.0000	VCT	PAWR	0.377166136
85	0.0000	0.375291617	0.687	0.527	0.0000	VCT	HSPD1	0.375291617
86	0.0000	0.356540783	0.47	0.251	0.0000	VCT	TLK1	0.356540783

87	0.0000	0.394336895	0.482	0.287	0.0000	VCT	GCSH	0.394336895
88	0.0000	0.405927744	0.684	0.382	0.0000	VCT	HMGA1	0.405927744
89	0.0000	0.355179828	0.407	0.201	0.0000	VCT	DLG5	0.355179828
90	0.0000	0.376012023	0.464	0.253	0.0000	VCT	DNMT1	0.376012023
91	0.0000	0.392230917	0.46	0.238	0.0000	VCT	UPP1	0.392230917
92	0.0000	0.3617706	0.512	0.304	0.0000	VCT	SNX2	0.3617706
93	0.0000	0.396093799	0.539	0.33	0.0000	VCT	TXNRD1	0.396093799
94	0.0000	0.439930239	0.597	0.415	0.0000	VCT	HIST1H4C	0.439930239
95	0.0000	0.350336654	0.544	0.366	0.0000	VCT	RAD23B	0.350336654
96	0.0000	0.371475216	0.452	0.259	0.0000	VCT	EIF4EBP1	0.371475216
97	0.0000	0.405928817	0.622	0.382	0.0000	VCT	GAS5	0.405928817
98	0.0000	0.358498861	0.572	0.399	0.0000	VCT	NASP	0.358498861
99	0.0000	0.358216679	0.393	0.205	0.0000	VCT	FLNB	0.358216679
100	0.0000	0.372192415	0.391	0.277	0.0000	VCT	IFI6	0.372192415
101	0.0000	0.803277848	0.765	0.341	0.0000	FB 1	COL18A1	0.803277848
102	0.0000	0.741011257	0.728	0.313	0.0000	FB 1	MEG3	0.741011257
103	0.0000	0.730544928	0.856	0.448	0.0000	FB 1	COL6A3	0.730544928
104	0.0000	0.664554366	0.827	0.482	0.0000	FB 1	DST	0.664554366
105	0.0000	0.638202749	0.891	0.508	0.0000	FB 1	COL6A2	0.638202749
106	0.0000	0.612717711	0.617	0.323	0.0000	FB 1	TBX3	0.612717711
107	0.0000	0.604713347	0.541	0.209	0.0000	FB 1	GOLGA8A	0.604713347
108	0.0000	0.601596081	0.939	0.642	0.0000	FB 1	XIST	0.601596081
109	0.0000	0.587829851	0.454	0.138	0.0000	FB 1	TSIX	0.587829851
110	0.0000	0.586733861	0.508	0.212	0.0000	FB 1	ABL2	0.586733861
111	0.0000	0.586022984	0.684	0.375	0.0000	FB 1	LRP1	0.586022984
112	0.0000	0.582670053	0.692	0.419	0.0000	FB 1	ADAMTS5	0.582670053
113	0.0000	0.576192128	0.648	0.387	0.0000	FB 1	IGF2R	0.576192128
114	0.0000	0.570216072	0.8	0.482	0.0000	FB 1	COL4A2	0.570216072
115	0.0000	0.565118025	0.849	0.477	0.0000	FB 1	COL6A1	0.565118025
116	0.0000	0.556014312	0.615	0.306	0.0000	FB 1	CCNL2	0.556014312
117	0.0000	0.548351702	0.497	0.211	0.0000	FB 1	THBS2	0.548351702
118	0.0000	0.54792038	0.617	0.303	0.0000	FB 1	PDGFRB	0.54792038
119	0.0000	0.540083576	0.565	0.276	0.0000	FB 1	FAP	0.540083576
120	0.0000	0.540057101	0.412	0.135	0.0000	FB 1	TMEM132A	0.540057101
121	0.0000	0.530802357	0.68	0.38	0.0000	FB 1	FTX	0.530802357
122	0.0000	0.521555482	0.83	0.536	0.0000	FB 1	FLNA	0.521555482
123	0.0000	0.50396457	0.617	0.328	0.0000	FB 1	SH3PXD2A	0.50396457
124	0.0000	0.497199881	0.596	0.297	0.0000	FB 1	DDR2	0.497199881
125	0.0000	0.49120801	0.415	0.164	0.0000	FB 1	AC020916.1	0.49120801
126	0.0000	0.489085813	0.362	0.112	0.0000	FB 1	COL7A1	0.489085813
127	0.0000	0.482010062	0.988	0.899	0.0000	FB 1	NEAT1	0.482010062
128	0.0000	0.480018245	0.561	0.284	0.0000	FB 1	ZEB2	0.480018245
129	0.0000	0.479810336	0.793	0.507	0.0000	FB 1	LAMB1	0.479810336
130	0.0000	0.478713737	0.783	0.524	0.0000	FB 1	COL4A1	0.478713737

131	0.0000	0.462294708	0.298	0.055	0.0000	FB 1	Z97200.1	0.462294708
132	0.0000	0.460323848	0.379	0.114	0.0000	FB 1	FAT1	0.460323848
133	0.0000	0.458205434	0.766	0.518	0.0000	FB 1	EGR1	0.458205434
134	0.0000	0.444040387	0.297	0.062	0.0000	FB 1	DNM1	0.444040387
135	0.0000	0.443831587	0.334	0.089	0.0000	FB 1	SGIP1	0.443831587
136	0.0000	0.437898093	0.317	0.08	0.0000	FB 1	SNHG14	0.437898093
137	0.0000	0.437151248	0.273	0.041	0.0000	FB 1	RFX8	0.437151248
138	0.0000	0.435584472	0.283	0.058	0.0000	FB 1	SPOCD1	0.435584472
139	0.0000	0.435405614	0.765	0.511	0.0000	FB 1	WSB1	0.435405614
140	0.0000	0.446899468	0.784	0.551	0.0000	FB 1	DYNC1H1	0.446899468
141	0.0000	0.416554367	0.401	0.153	0.0000	FB 1	ANKRD36C	0.416554367
142	0.0000	0.439020834	0.456	0.214	0.0000	FB 1	EMILIN1	0.439020834
143	0.0000	0.442555868	0.702	0.447	0.0000	FB 1	VMP1	0.442555868
144	0.0000	0.427181769	0.348	0.128	0.0000	FB 1	AC016831.1	0.427181769
145	0.0000	0.383338077	0.335	0.121	0.0000	FB 1	NR2F2-AS1	0.383338077
146	0.0000	0.44137406	0.594	0.336	0.0000	FB 1	TEAD1	0.44137406
147	0.0000	0.417803368	0.354	0.126	0.0000	FB 1	NAV2	0.417803368
148	0.0000	0.461909211	0.671	0.419	0.0000	FB 1	MAP4K4	0.461909211
149	0.0000	0.435379267	0.546	0.296	0.0000	FB 1	PXDN	0.435379267
150	0.0000	0.42574178	0.564	0.312	0.0000	FB 1	ECE1	0.42574178
151	0.0000	0.423297222	0.807	0.598	0.0000	FB 1	MACF1	0.423297222
152	0.0000	0.416471331	0.634	0.387	0.0000	FB 1	CTTN	0.416471331
153	0.0000	0.391869695	0.795	0.551	0.0000	FB 1	ARGLU1	0.391869695
154	0.0000	0.477263893	0.726	0.5	0.0000	FB 1	RND3	0.477263893
155	0.0000	0.450216379	0.468	0.234	0.0000	FB 1	PLEKHH2	0.450216379
156	0.0000	0.391509748	0.331	0.122	0.0000	FB 1	MIR222HG	0.391509748
157	0.0000	0.439992605	0.474	0.232	0.0000	FB 1	LYST	0.439992605
158	0.0000	0.438705922	0.634	0.371	0.0000	FB 1	MAP1B	0.438705922
159	0.0000	0.439396462	0.719	0.454	0.0000	FB 1	PRRX1	0.439396462
160	0.0000	0.447709422	0.556	0.311	0.0000	FB 1	NFKBIZ	0.447709422
161	0.0000	0.426824493	0.533	0.295	0.0000	FB 1	PCNX4	0.426824493
162	0.0000	0.494453052	0.608	0.346	0.0000	FB 1	PHLDA1	0.494453052
163	0.0000	0.406653364	0.376	0.16	0.0000	FB 1	RASSF8-AS1	0.406653364
164	0.0000	0.471201243	0.606	0.368	0.0000	FB 1	ATP13A3	0.471201243
165	0.0000	0.429798611	0.609	0.359	0.0000	FB 1	NRP1	0.429798611
166	0.0000	0.38640408	0.339	0.134	0.0000	FB 1	RHBDF1	0.38640408
167	0.0000	0.417631768	0.746	0.531	0.0000	FB 1	MMP14	0.417631768
168	0.0000	0.416270201	0.48	0.233	0.0000	FB 1	CDH13	0.416270201
169	0.0000	0.426377181	0.545	0.312	0.0000	FB 1	GPATCH2L	0.426377181
170	0.0000	0.450767864	0.666	0.426	0.0000	FB 1	SOX4	0.450767864
171	0.0000	0.418833046	0.41	0.195	0.0000	FB 1	AHNAK2	0.418833046
172	0.0000	0.456795927	0.557	0.327	0.0000	FB 1	TUT7	0.456795927
173	0.0000	0.395340841	0.405	0.191	0.0000	FB 1	SDK1	0.395340841
174	0.0000	0.405749735	0.406	0.194	0.0000	FB 1	PLA2R1	0.405749735

175	0.0000	0.391618543	0.415	0.193	0.0000	FB 1	BTAF1	0.391618543
176	0.0000	0.388479719	0.764	0.566	0.0000	FB 1	MCL1	0.388479719
177	0.0000	0.394220521	0.707	0.471	0.0000	FB 1	NKTR	0.394220521
178	0.0000	0.407771156	0.278	0.091	0.0000	FB 1	SOX11	0.407771156
179	0.0000	0.423183314	0.45	0.225	0.0000	FB 1	DCBLD2	0.423183314
180	0.0000	0.37700207	0.582	0.337	0.0000	FB 1	KANK2	0.37700207
181	0.0000	0.381640913	0.337	0.137	0.0000	FB 1	PLK3	0.381640913
182	0.0000	0.384337175	0.474	0.262	0.0000	FB 1	MLXIP	0.384337175
183	0.0000	0.387161866	0.516	0.297	0.0000	FB 1	CSNK1E	0.387161866
184	0.0000	0.393961596	0.726	0.477	0.0000	FB 1	NR2F2	0.393961596
185	0.0000	0.383791779	0.462	0.24	0.0000	FB 1	BACH1	0.383791779
186	0.0000	0.381767024	0.302	0.123	0.0000	FB 1	MEG8	0.381767024
187	0.0000	0.388579371	0.569	0.353	0.0000	FB 1	SH3PXD2B	0.388579371
188	0.0000	0.446857328	0.393	0.183	0.0000	FB 1	ITGA2	0.446857328
189	0.0000	0.378434441	0.669	0.459	0.0000	FB 1	ZBTB38	0.378434441
190	0.0000	0.379526257	0.715	0.446	0.0000	FB 1	MFGE8	0.379526257
191	0.0000	0.376740341	0.579	0.351	0.0000	FB 1	AKAP12	0.376740341
192	0.0000	0.378436804	0.846	0.597	0.0000	FB 1	CALD1	0.378436804
193	0.0000	0.393868354	0.428	0.206	0.0000	FB 1	PALM2-AKAP2	0.393868354
194	0.0000	0.424465013	0.608	0.381	0.0000	FB 1	KCNQ1OT1	0.424465013
195	0.0000	0.4524026	0.641	0.414	0.0000	FB 1	COL1A2	0.4524026
196	0.0000	0.399968959	0.452	0.26	0.0000	FB 1	TNFAIP3	0.399968959
197	0.0000	0.434167513	0.31	0.149	0.0000	FB 1	NOTCH3	0.434167513
198	0.0000	0.37587989	0.515	0.303	0.0000	FB 1	CCND1	0.37587989
199	0.0000	0.435399715	0.167	0.057	0.0000	FB 1	RGS5	0.435399715
200	0.0000	0.377183835	0.525	0.427	0.0000	FB 1	COL1A1	0.377183835
201	0.0000	0.669756929	0.495	0.216	0.0000	FB 2	DKK1	0.669756929
202	0.0000	0.66107936	0.686	0.5	0.0000	FB 2	CFD	0.66107936
203	0.0000	0.657516029	0.542	0.186	0.0000	FB 2	RAMP1	0.657516029
204	0.0000	0.638192914	0.775	0.476	0.0000	FB 2	APOD	0.638192914
205	0.0000	0.611867469	0.686	0.408	0.0000	FB 2	C1R	0.611867469
206	0.0000	0.604279017	0.571	0.244	0.0000	FB 2	IGFBP6	0.604279017
207	0.0000	0.602052163	0.487	0.119	0.0000	FB 2	HSPB6	0.602052163
208	0.0000	0.598371326	0.62	0.36	0.0000	FB 2	PCOLCE	0.598371326
209	0.0000	0.595257736	0.549	0.183	0.0000	FB 2	PLPP1	0.595257736
210	0.0000	0.592536447	0.513	0.166	0.0000	FB 2	TMEM45A	0.592536447
211	0.0000	0.56930574	0.637	0.382	0.0000	FB 2	TCEAL4	0.56930574
212	0.0000	0.554315835	0.667	0.355	0.0000	FB 2	C1S	0.554315835
213	0.0000	0.550401065	0.572	0.211	0.0000	FB 2	CPXM1	0.550401065
214	0.0000	0.547319208	0.711	0.385	0.0000	FB 2	SELENOM	0.547319208
215	0.0000	0.547189122	0.513	0.227	0.0000	FB 2	SERPINF1	0.547189122
216	0.0000	0.545998382	0.501	0.188	0.0000	FB 2	PTN	0.545998382
217	0.0000	0.544043607	0.465	0.118	0.0000	FB 2	SCARA5	0.544043607
218	0.0000	0.534393151	0.631	0.32	0.0000	FB 2	SERPING1	0.534393151

219	0.0000	0.530123817	0.775	0.688	0.0000	FB 2	PTGDS	0.530123817
220	0.0000	0.527575872	0.507	0.156	0.0000	FB 2	ISLR	0.527575872
221	0.0000	0.519200812	0.584	0.26	0.0000	FB 2	CD248	0.519200812
222	0.0000	0.5123974	0.677	0.368	0.0000	FB 2	MYL9	0.5123974
223	0.0000	0.510152334	0.583	0.376	0.0000	FB 2	S100A4	0.510152334
224	0.0000	0.506719021	0.584	0.245	0.0000	FB 2	RORB	0.506719021
225	0.0000	0.505452556	0.501	0.218	0.0000	FB 2	APCDD1	0.505452556
226	0.0000	0.497858305	0.53	0.182	0.0000	FB 2	SULF2	0.497858305
227	0.0000	0.497335756	0.56	0.225	0.0000	FB 2	CCDC80	0.497335756
228	0.0000	0.492928746	0.471	0.108	0.0000	FB 2	OLFML3	0.492928746
229	0.0000	0.49279116	0.778	0.512	0.0000	FB 2	RBP1	0.49279116
230	0.0000	0.490050329	0.554	0.233	0.0000	FB 2	PRSS23	0.490050329
231	0.0000	0.486553638	0.493	0.141	0.0000	FB 2	OSR2	0.486553638
232	0.0000	0.486120028	0.819	0.718	0.0000	FB 2	IGFBP2	0.486120028
233	0.0000	0.485247175	0.553	0.207	0.0000	FB 2	HAND2	0.485247175
234	0.0000	0.478196245	0.787	0.601	0.0000	FB 2	APOE	0.478196245
235	0.0000	0.467503121	0.588	0.28	0.0000	FB 2	SPON2	0.467503121
236	0.0000	0.466260894	0.512	0.156	0.0000	FB 2	TCEAL3	0.466260894
237	0.0000	0.465973602	0.625	0.304	0.0000	FB 2	MDK	0.465973602
238	0.0000	0.465800479	0.563	0.267	0.0000	FB 2	TWISTNB	0.465800479
239	0.0000	0.4640666	0.299	0.066	0.0000	FB 2	PLA2G2A	0.4640666
240	0.0000	0.462532694	0.736	0.427	0.0000	FB 2	TPM2	0.462532694
241	0.0000	0.462325521	0.402	0.111	0.0000	FB 2	IGF1	0.462325521
242	0.0000	0.458676505	0.682	0.396	0.0000	FB 2	BASP1	0.458676505
243	0.0000	0.457427628	0.503	0.157	0.0000	FB 2	HAND2-AS1	0.457427628
244	0.0000	0.455556581	0.789	0.716	0.0000	FB 2	DCN	0.455556581
245	0.0000	0.452218478	0.443	0.094	0.0000	FB 2	PLAC9	0.452218478
246	0.0000	0.452116522	0.555	0.187	0.0000	FB 2	PDGFRA	0.452116522
247	0.0000	0.452114642	0.536	0.187	0.0000	FB 2	MXRA8	0.452114642
248	0.0000	0.450993486	0.557	0.227	0.0000	FB 2	FBLN2	0.450993486
249	0.0000	0.447891684	0.634	0.313	0.0000	FB 2	MGST1	0.447891684
250	0.0000	0.446459741	0.648	0.326	0.0000	FB 2	DPYSL2	0.446459741
251	0.0000	0.444270542	0.607	0.293	0.0000	FB 2	MEG3	0.444270542
252	0.0000	0.440805824	0.581	0.302	0.0000	FB 2	GSN	0.440805824
253	0.0000	0.437476606	0.553	0.18	0.0000	FB 2	MEDAG	0.437476606
254	0.0000	0.433726696	0.698	0.403	0.0000	FB 2	MFGE8	0.433726696
255	0.0000	0.432298809	0.494	0.201	0.0000	FB 2	MGP	0.432298809
256	0.0000	0.429860265	0.49	0.189	0.0000	FB 2	CRISPLD2	0.429860265
257	0.0000	0.425087941	0.516	0.208	0.0000	FB 2	CRYAB	0.425087941
258	0.0000	0.424303925	0.541	0.195	0.0000	FB 2	THY1	0.424303925
259	0.0000	0.41510276	0.442	0.105	0.0000	FB 2	TMEM37	0.41510276
260	0.0000	0.413765925	0.698	0.516	0.0000	FB 2	HSPB1	0.413765925
261	0.0000	0.412424199	0.846	0.67	0.0000	FB 2	LGALS3	0.412424199
262	0.0000	0.412101274	0.441	0.108	0.0000	FB 2	SPOCK1	0.412101274

263	0.0000	0.411757851	0.46	0.108	0.0000	FB 2	COPZ2	0.411757851
264	0.0000	0.410460869	0.611	0.304	0.0000	FB 2	REXO2	0.410460869
265	0.0000	0.409522799	0.606	0.331	0.0000	FB 2	NID1	0.409522799
266	0.0000	0.40897205	0.526	0.191	0.0000	FB 2	COX7A1	0.40897205
267	0.0000	0.405540241	0.766	0.643	0.0000	FB 2	CST3	0.405540241
268	0.0000	0.402950088	0.578	0.305	0.0000	FB 2	PDLIM1	0.402950088
269	0.0000	0.40065645	0.524	0.208	0.0000	FB 2	ID4	0.40065645
270	0.0000	0.398252192	0.656	0.34	0.0000	FB 2	S100A16	0.398252192
271	0.0000	0.396614919	0.76	0.591	0.0000	FB 2	CALD1	0.396614919
272	0.0000	0.395982792	0.561	0.225	0.0000	FB 2	PDLIM2	0.395982792
273	0.0000	0.39391968	0.878	0.712	0.0000	FB 2	TXN	0.39391968
274	0.0000	0.393001161	0.722	0.444	0.0000	FB 2	EMP3	0.393001161
275	0.0000	0.391086217	0.779	0.528	0.0000	FB 2	TUBA1A	0.391086217
276	0.0000	0.390255009	0.483	0.133	0.0000	FB 2	EFEMP2	0.390255009
277	0.0000	0.389868046	0.524	0.199	0.0000	FB 2	CDH11	0.389868046
278	0.0000	0.388096227	0.635	0.341	0.0000	FB 2	NNMT	0.388096227
279	0.0000	0.388062004	0.441	0.167	0.0000	FB 2	BTBD3	0.388062004
280	0.0000	0.386375215	0.525	0.193	0.0000	FB 2	PPIC	0.386375215
281	0.0000	0.380840756	0.468	0.127	0.0000	FB 2	ADRA2C	0.380840756
282	0.0000	0.380520902	0.632	0.291	0.0000	FB 2	MXRA7	0.380520902
283	0.0000	0.380372749	0.652	0.364	0.0000	FB 2	S100A13	0.380372749
284	0.0000	0.379861828	0.559	0.224	0.0000	FB 2	RRAS	0.379861828
285	0.0000	0.379738739	0.806	0.715	0.0000	FB 2	CD81	0.379738739
286	0.0000	0.379034085	0.55	0.207	0.0000	FB 2	DDAH2	0.379034085
287	0.0000	0.378663407	0.569	0.221	0.0000	FB 2	SEMA5A	0.378663407
288	0.0000	0.377909337	0.405	0.135	0.0000	FB 2	PAPPA	0.377909337
289	0.0000	0.3767056	0.524	0.228	0.0000	FB 2	IRS2	0.3767056
290	0.0000	0.375556193	0.556	0.267	0.0000	FB 2	PLPP3	0.375556193
291	0.0000	0.375405105	0.337	0.099	0.0000	FB 2	CHRDL1	0.375405105
292	0.0000	0.374420267	0.498	0.159	0.0000	FB 2	C20orf27	0.374420267
293	0.0000	0.374027674	0.54	0.267	0.0000	FB 2	SPTSSA	0.374027674
294	0.0000	0.375916136	0.703	0.444	0.0000	FB 2	PTMS	0.375916136
295	0.0000	0.54509172	0.495	0.296	0.0000	FB 2	IGFBP5	0.54509172
296	0.0000	0.422213838	0.607	0.405	0.0000	FB 2	IGFBP4	0.422213838
297	0.0000	0.384289384	0.758	0.682	0.0000	FB 2	LUM	0.384289384
298	0.0000	0.398797284	0.438	0.212	0.0000	FB 2	TAGLN	0.398797284
299	0.0000	0.378670351	0.64	0.446	0.0000	FB 2	VCAN	0.378670351
300	0.0000	0.430797074	0.558	0.373	0.0000	FB 2	SPARCL1	0.430797074
301	0.0000	1.068513096	0.995	0.71	0.0000	SCT	CGA	1.068513096
302	0.0000	1.054258603	0.709	0.36	0.0000	SCT	KISS1	1.054258603
303	0.0000	0.964125263	0.769	0.455	0.0000	SCT	CGB3	0.964125263
304	0.0000	0.679217903	0.318	0.064	0.0000	SCT	PLAC4	0.679217903
305	0.0000	0.753420895	0.522	0.272	0.0000	SCT	S100P	0.753420895
306	0.0000	0.704395165	0.59	0.396	0.0000	SCT	GDF15	0.704395165

307	0.0000	0.657085064	0.351	0.131	0.0000	SCT	HSD17B1	0.657085064
308	0.0000	0.682887057	0.572	0.385	0.0000	SCT	TFPI2	0.682887057
309	0.0000	0.519092694	0.77	0.693	0.0000	SCT	PAEP	0.519092694
310	0.0000	0.621488417	0.372	0.174	0.0000	SCT	PSG3	0.621488417
311	0.0000	0.548550569	0.257	0.088	0.0000	SCT	LEP	0.548550569
312	0.0000	0.427867919	0.193	0.055	0.0000	SCT	CSH2	0.427867919
313	0.0000	0.428174712	0.227	0.073	0.0000	SCT	S100A9	0.428174712
314	0.0000	0.514927396	0.41	0.248	0.0000	SCT	CSH1	0.514927396
315	0.0000	0.421755629	0.255	0.112	0.0000	SCT	CLIC3	0.421755629
316	0.0000	0.419572695	0.562	0.456	0.0000	SCT	KRT18	0.419572695
317	0.0000	0.402835583	0.237	0.114	0.0000	SCT	PSG5	0.402835583
318	0.0000	0.322225278	0.205	0.087	0.0000	SCT	HOPX	0.322225278
319	0.0000	0.34244469	0.222	0.106	0.0000	SCT	PSG2	0.34244469
320	0.0000	0.392073429	0.42	0.331	0.0000	SCT	KRT19	0.392073429
321	0.0000	0.414645387	0.224	0.13	0.0000	SCT	PSG9	0.414645387
322	0.0000	0.260178362	0.156	0.072	0.0000	SCT	CCSAP	0.260178362
323	0.0000	0.355250497	0.43	0.336	0.0000	SCT	KRT8	0.355250497
324	0.0000	0.35657276	0.178	0.096	0.0000	SCT	PSG1	0.35657276
325	0.0000	0.311346387	0.16	0.081	0.0000	SCT	PSG4	0.311346387
326	0.0000	0.296878912	0.484	0.439	0.0000	SCT	MTATP6P1	0.296878912
327	0.0000	0.500398713	0.133	0.07	0.0000	SCT	HBA1	0.500398713
328	0.0000	0.287805497	0.194	0.128	0.0000	SCT	TACC2	0.287805497
329	0.0000	0.637255127	0.229	0.183	0.0000	SCT	HBB	0.637255127
330	0.0000	0.608530046	0.21	0.162	0.0000	SCT	HBA2	0.608530046
331	0.0000	0.289442676	0.205	0.151	0.0000	SCT	PAGE4	0.289442676
332	0.0000	0.255453908	0.171	0.123	0.0000	SCT	ADAM12	0.255453908
333	0.0000	0.277907936	0.185	0.15	0.0000	SCT	CYP11A1	0.277907936
334	0.0000	0.288181193	0.209	0.18	0.0000	SCT	FLT1	0.288181193
335	0.0000	0.31564646	0.246	0.234	0.0000	SCT	FDX1	0.31564646
336	0.0000	0.261778059	0.288	0.299	0.0159	SCT	VGLL3	0.261778059
337	0.0000	0.329141199	0.295	0.315	0.0964	SCT	GPX3	0.329141199
338	0.0000	1.323945314	0.645	0.278	0.0000	T & NK	GNLY	1.323945314
339	0.0000	1.255566046	0.75	0.095	0.0000	T & NK	PTPRC	1.255566046
340	0.0000	1.228438969	0.536	0.076	0.0000	T & NK	NKG7	1.228438969
341	0.0000	1.205052623	0.518	0.043	0.0000	T & NK	CCL5	1.205052623
342	0.0000	1.06782149	0.491	0.032	0.0000	T & NK	GZMA	1.06782149
343	0.0000	0.984062414	0.491	0.096	0.0000	T & NK	CCL4	0.984062414
344	0.0000	0.983497297	0.559	0.058	0.0000	T & NK	CORO1A	0.983497297
345	0.0000	0.936247976	0.46	0.048	0.0000	T & NK	IL2RB	0.936247976
346	0.0000	0.884091206	0.424	0.019	0.0000	T & NK	PRF1	0.884091206
347	0.0000	0.867770893	0.542	0.158	0.0000	T & NK	SRGN	0.867770893
348	0.0000	0.866549159	0.384	0.022	0.0000	T & NK	CTSW	0.866549159
349	0.0000	0.84886369	0.447	0.054	0.0000	T & NK	ARAP2	0.84886369
350	0.0000	0.841809571	0.371	0.024	0.0000	T & NK	KLRD1	0.841809571

351	0.0000	0.83992569	0.558	0.25	0.0000	T & NK	IL32	0.83992569
352	0.0000	0.839855218	0.426	0.017	0.0000	T & NK	CST7	0.839855218
353	0.0000	0.83127953	0.409	0.026	0.0000	T & NK	HCST	0.83127953
354	0.0000	0.830375692	0.394	0.068	0.0000	T & NK	ITGAX	0.830375692
355	0.0000	0.822744633	0.461	0.048	0.0000	T & NK	RAC2	0.822744633
356	0.0000	0.814554106	0.346	0.017	0.0000	T & NK	KLRC1	0.814554106
357	0.0000	0.803388682	0.567	0.25	0.0000	T & NK	EVL	0.803388682
358	0.0000	0.798510422	0.423	0.036	0.0000	T & NK	SLA	0.798510422
359	0.0000	0.779343859	0.413	0.044	0.0000	T & NK	CD52	0.779343859
360	0.0000	0.773950515	0.374	0.015	0.0000	T & NK	SAMD3	0.773950515
361	0.0000	0.769433777	0.384	0.016	0.0000	T & NK	CD2	0.769433777
362	0.0000	0.76568394	0.343	0.028	0.0000	T & NK	NCAM1	0.76568394
363	0.0000	0.755868453	0.475	0.124	0.0000	T & NK	STK17B	0.755868453
364	0.0000	0.743119465	0.332	0.013	0.0000	T & NK	KLRB1	0.743119465
365	0.0000	0.7430907	0.42	0.072	0.0000	T & NK	ACAP1	0.7430907
366	0.0000	0.739465723	0.486	0.109	0.0000	T & NK	FYB1	0.739465723
367	0.0000	0.73590552	0.633	0.337	0.0000	T & NK	LCP1	0.73590552
368	0.0000	0.73226243	0.378	0.025	0.0000	T & NK	CD69	0.73226243
369	0.0000	0.732224425	0.357	0.018	0.0000	T & NK	CD7	0.732224425
370	0.0000	0.727915906	0.332	0.072	0.0000	T & NK	TTN	0.727915906
371	0.0000	0.722777344	0.369	0.013	0.0000	T & NK	CD96	0.722777344
372	0.0000	0.717551576	0.383	0.034	0.0000	T & NK	ARHGAP30	0.717551576
373	0.0000	0.716127806	0.383	0.023	0.0000	T & NK	ITGAL	0.716127806
374	0.0000	0.703356884	0.362	0.015	0.0000	T & NK	CD247	0.703356884
375	0.0000	0.693972039	0.333	0.065	0.0000	T & NK	TYROBP	0.693972039
376	0.0000	0.69248565	0.359	0.028	0.0000	T & NK	IKZF3	0.69248565
377	0.0000	0.68962422	0.358	0.087	0.0000	T & NK	CCL3	0.68962422
378	0.0000	0.683894101	0.373	0.059	0.0000	T & NK	SEMA4D	0.683894101
379	0.0000	0.680753344	0.287	0.015	0.0000	T & NK	GZMB	0.680753344
380	0.0000	0.678070037	0.347	0.021	0.0000	T & NK	RUNX3	0.678070037
381	0.0000	0.655203767	0.352	0.013	0.0000	T & NK	CD3E	0.655203767
382	0.0000	0.64336404	0.441	0.128	0.0000	T & NK	ARHGDIB	0.64336404
383	0.0000	0.637231041	0.324	0.019	0.0000	T & NK	TRBC2	0.637231041
384	0.0000	0.634925785	0.341	0.042	0.0000	T & NK	DOCK10	0.634925785
385	0.0000	0.633175675	0.357	0.04	0.0000	T & NK	CD48	0.633175675
386	0.0000	0.623592614	0.359	0.051	0.0000	T & NK	LCP2	0.623592614
387	0.0000	0.621170853	0.407	0.126	0.0000	T & NK	CELF2	0.621170853
388	0.0000	0.61187025	0.26	0.012	0.0000	T & NK	TRDC	0.61187025
389	0.0000	0.609445895	0.306	0.011	0.0000	T & NK	PYHIN1	0.609445895
390	0.0000	0.606858624	0.256	0.007	0.0000	T & NK	XCL2	0.606858624
391	0.0000	0.600666207	0.336	0.031	0.0000	T & NK	IKZF1	0.600666207
392	0.0000	0.591001236	0.233	0.009	0.0000	T & NK	XCL1	0.591001236
393	0.0000	0.582340397	0.274	0.015	0.0000	T & NK	TRAC	0.582340397
394	0.0000	0.575870664	0.236	0.011	0.0000	T & NK	ITGAD	0.575870664

395	0.0000	0.562472177	0.336	0.069	0.0000	T & NK	DOCK8	0.562472177
396	0.0000	0.552740982	0.317	0.047	0.0000	T & NK	GIMAP7	0.552740982
397	0.0000	0.536606044	0.281	0.023	0.0000	T & NK	PTPN7	0.536606044
398	0.0000	0.535805922	0.361	0.08	0.0000	T & NK	LAPTM5	0.535805922
399	0.0000	0.534381795	0.291	0.019	0.0000	T & NK	TRAF3IP3	0.534381795
400	0.0000	0.534097787	0.283	0.016	0.0000	T & NK	IL2RG	0.534097787
401	0.0000	0.532548628	0.279	0.016	0.0000	T & NK	SPN	0.532548628
402	0.0000	0.531984182	0.249	0.01	0.0000	T & NK	CD3D	0.531984182
403	0.0000	0.530967608	0.314	0.053	0.0000	T & NK	IQGAP2	0.530967608
404	0.0000	0.516828093	0.312	0.066	0.0000	T & NK	PRKCH	0.516828093
405	0.0000	0.513097602	0.242	0.03	0.0000	T & NK	LTB	0.513097602
406	0.0000	0.496531863	0.238	0.014	0.0000	T & NK	ARHGAP9	0.496531863
407	0.0000	0.493648328	0.308	0.054	0.0000	T & NK	CD53	0.493648328
408	0.0000	0.486036204	0.282	0.043	0.0000	T & NK	SORL1	0.486036204
409	0.0000	0.480780345	0.255	0.02	0.0000	T & NK	TMC8	0.480780345
410	0.0000	0.478849938	0.29	0.05	0.0000	T & NK	GMFG	0.478849938
411	0.0000	0.473874861	0.248	0.027	0.0000	T & NK	CCDC88C	0.473874861
412	0.0000	0.470004018	0.313	0.066	0.0000	T & NK	CD37	0.470004018
413	0.0000	0.469086164	0.21	0.008	0.0000	T & NK	TRBC1	0.469086164
414	0.0000	0.466112396	0.276	0.049	0.0000	T & NK	GIMAP4	0.466112396
415	0.0000	0.644593352	0.39	0.118	0.0000	T & NK	LSP1	0.644593352
416	0.0000	0.509269333	0.267	0.058	0.0000	T & NK	APOBEC3G	0.509269333
417	0.0000	0.471381179	0.281	0.066	0.0000	T & NK	ANKRD44	0.471381179
418	0.0000	0.513125193	0.309	0.079	0.0000	T & NK	HOPX	0.513125193
419	0.0000	0.524479085	0.245	0.05	0.0000	T & NK	FCER1G	0.524479085
420	0.0000	0.523491678	0.358	0.11	0.0000	T & NK	MBP	0.523491678
421	0.0000	0.580560004	0.425	0.171	0.0000	T & NK	WIPF1	0.580560004
422	0.0000	0.472165726	0.274	0.072	0.0000	T & NK	PARP8	0.472165726
423	0.0000	0.696132715	0.469	0.228	0.0000	T & NK	GNPTAB	0.696132715
424	0.0000	0.568757924	0.434	0.187	0.0000	T & NK	STK17A	0.568757924
425	0.0000	0.512368684	0.347	0.125	0.0000	T & NK	AC245297.3	0.512368684
426	0.0000	0.528125516	0.709	0.525	0.0000	T & NK	RNF213	0.528125516
427	0.0000	0.530171151	0.42	0.187	0.0000	T & NK	ARL4C	0.530171151
428	0.0000	0.517624752	0.351	0.142	0.0000	T & NK	AKNA	0.517624752
429	0.0000	0.567763341	0.375	0.164	0.0000	T & NK	ARHGEF1	0.567763341
430	0.0000	0.584956959	0.486	0.279	0.0000	T & NK	ID2	0.584956959
431	0.0000	0.475302684	0.262	0.086	0.0000	T & NK	NR4A2	0.475302684
432	0.0000	0.520073513	0.459	0.256	0.0000	T & NK	STK4	0.520073513
433	0.0000	0.517542612	0.421	0.229	0.0000	T & NK	SYNRG	0.517542612
434	0.0000	0.477007432	0.541	0.388	0.0000	T & NK	MBNL1	0.477007432
435	0.0000	0.558968436	0.419	0.26	0.0000	T & NK	HERC1	0.558968436
436	0.0000	0.476699295	0.369	0.21	0.0000	T & NK	RBL2	0.476699295
437	0.0000	0.495127748	0.454	0.343	0.0000	T & NK	COTL1	0.495127748
438	0.0000	1.397717867	0.711	0.116	0.0000	EVT	DIO2	1.397717867

439	0.0000	1.37323885	0.718	0.132	0.0000	EVT	NOTUM	1.37323885
440	0.0000	1.301049493	0.705	0.106	0.0000	EVT	HTRA4	1.301049493
441	0.0000	1.161223126	0.63	0.082	0.0000	EVT	HLA-G	1.161223126
442	0.0000	1.128992264	0.601	0.07	0.0000	EVT	ISM2	1.128992264
443	0.0000	1.127425389	0.661	0.096	0.0000	EVT	PGF	1.127425389
444	0.0000	0.949290331	0.581	0.078	0.0000	EVT	CCNE1	0.949290331
445	0.0000	0.818622524	0.452	0.04	0.0000	EVT	ASCL2	0.818622524
446	0.0000	0.676086012	0.41	0.032	0.0000	EVT	MYCN	0.676086012
447	0.0000	0.611399365	0.401	0.021	0.0000	EVT	GCM1	0.611399365
448	0.0000	0.595049811	0.372	0.016	0.0000	EVT	MYCNUT	0.595049811
449	0.0000	0.51626397	0.363	0.018	0.0000	EVT	N4BP3	0.51626397
450	0.0000	0.575985454	0.352	0.029	0.0000	EVT	CSF2RB	0.575985454
451	0.0000	0.73238389	0.421	0.044	0.0000	EVT	SLCO4A1	0.73238389
452	0.0000	1.11943119	0.645	0.118	0.0000	EVT	ADAM12	1.11943119
453	0.0000	0.638821544	0.425	0.049	0.0000	EVT	GJA5	0.638821544
454	0.0000	0.542395099	0.357	0.037	0.0000	EVT	PLAC8	0.542395099
455	0.0000	1.006088178	0.526	0.093	0.0000	EVT	HPGD	1.006088178
456	0.0000	0.864972123	0.529	0.093	0.0000	EVT	MFAP5	0.864972123
457	0.0000	0.496451607	0.348	0.039	0.0000	EVT	NR6A1	0.496451607
458	0.0000	0.514215665	0.403	0.053	0.0000	EVT	PKP3	0.514215665
459	0.0000	0.667694685	0.434	0.067	0.0000	EVT	PRSS8	0.667694685
460	0.0000	0.495959603	0.383	0.051	0.0000	EVT	GRHL1	0.495959603
461	0.0000	1.194991951	0.694	0.211	0.0000	EVT	PTPRF	1.194991951
462	0.0000	0.51312495	0.33	0.038	0.0000	EVT	LINC01949	0.51312495
463	0.0000	0.736165736	0.502	0.093	0.0000	EVT	DSC2	0.736165736
464	0.0000	0.579991264	0.449	0.073	0.0000	EVT	IGF2BP3	0.579991264
465	0.0000	0.841340148	0.599	0.136	0.0000	EVT	GATA3	0.841340148
466	0.0000	0.557592872	0.403	0.063	0.0000	EVT	ASAP3	0.557592872
467	0.0000	0.535533645	0.425	0.067	0.0000	EVT	LIN28B	0.535533645
468	0.0000	0.555003153	0.363	0.052	0.0000	EVT	AIF1L	0.555003153
469	0.0000	0.613471692	0.456	0.078	0.0000	EVT	TFAP2A	0.613471692
470	0.0000	0.495457353	0.39	0.058	0.0000	EVT	HSD3B1	0.495457353
471	0.0000	0.652978525	0.374	0.057	0.0000	EVT	ADAM19	0.652978525
472	0.0000	0.62518444	0.427	0.071	0.0000	EVT	TENM3	0.62518444
473	0.0000	0.644974154	0.419	0.071	0.0000	EVT	EFNA1	0.644974154
474	0.0000	0.877155912	0.883	0.61	0.0000	EVT	TPM1	0.877155912
475	0.0000	1.134154835	0.828	0.41	0.0000	EVT	CDKN1C	1.134154835
476	0.0000	0.909744309	0.458	0.093	0.0000	EVT	COL27A1	0.909744309
477	0.0000	0.934593652	0.608	0.165	0.0000	EVT	HEG1	0.934593652
478	0.0000	0.993552113	0.643	0.209	0.0000	EVT	ARID3A	0.993552113
479	0.0000	0.531447494	0.396	0.067	0.0000	EVT	PKP2	0.531447494
480	0.0000	1.017184453	0.511	0.122	0.0000	EVT	LAIR2	1.017184453
481	0.0000	0.553485231	0.39	0.065	0.0000	EVT	ERVH48-1	0.553485231
482	0.0000	0.516894052	0.379	0.062	0.0000	EVT	NLRP2	0.516894052

483	0.0000	0.655165047	0.447	0.093	0.0000	EVT	ERBB2	0.655165047
484	0.0000	0.885770283	0.57	0.154	0.0000	EVT	MCAM	0.885770283
485	0.0000	1.163728986	0.667	0.259	0.0000	EVT	QSOX1	1.163728986
486	0.0000	1.010983723	0.672	0.238	0.0000	EVT	KRT7	1.010983723
487	0.0000	0.935964259	0.771	0.339	0.0000	EVT	KRT8	0.935964259
488	0.0000	0.929555331	0.617	0.211	0.0000	EVT	FLNB	0.929555331
489	0.0000	1.011212493	0.738	0.334	0.0000	EVT	KRT19	1.011212493
490	0.0000	0.506880615	0.421	0.088	0.0000	EVT	F11R	0.506880615
491	0.0000	0.861753566	0.621	0.199	0.0000	EVT	DSP	0.861753566
492	0.0000	0.555337874	0.377	0.075	0.0000	EVT	IL1RAP	0.555337874
493	0.0000	0.528728178	0.414	0.09	0.0000	EVT	TET3	0.528728178
494	0.0000	0.760772298	0.52	0.147	0.0000	EVT	LIFR	0.760772298
495	0.0000	1.151749055	0.581	0.225	0.0000	EVT	FSTL3	1.151749055
496	0.0000	0.530151226	0.348	0.072	0.0000	EVT	CDH5	0.530151226
497	0.0000	0.624947362	0.493	0.142	0.0000	EVT	DOCK5	0.624947362
498	0.0000	0.504644155	0.407	0.098	0.0000	EVT	DSG2	0.504644155
499	0.0000	0.519540103	0.383	0.092	0.0000	EVT	LRRC32	0.519540103
500	0.0000	0.793777747	0.654	0.29	0.0000	EVT	VGLL3	0.793777747
501	0.0000	0.604417584	0.482	0.149	0.0000	EVT	NAV1	0.604417584
502	0.0000	0.727689068	0.584	0.216	0.0000	EVT	SERPINE2	0.727689068
503	0.0000	0.578005628	0.465	0.142	0.0000	EVT	GATA2	0.578005628
504	0.0000	0.762870447	0.643	0.319	0.0000	EVT	NRIP1	0.762870447
505	0.0000	0.517891363	0.412	0.118	0.0000	EVT	TNS3	0.517891363
506	0.0000	0.714317827	0.542	0.204	0.0000	EVT	PHLDA2	0.714317827
507	0.0000	0.808116616	0.5	0.188	0.0000	EVT	SLC16A3	0.808116616
508	0.0000	0.696635764	0.813	0.463	0.0000	EVT	KRT18	0.696635764
509	0.0000	0.778707551	0.586	0.294	0.0000	EVT	ITGA5	0.778707551
510	0.0000	0.534568727	0.388	0.122	0.0000	EVT	SEMA4C	0.534568727
511	0.0000	0.53000054	0.401	0.121	0.0000	EVT	XAGE2	0.53000054
512	0.0000	0.677202629	0.764	0.572	0.0000	EVT	COL4A1	0.677202629
513	0.0000	0.772476839	0.46	0.177	0.0000	EVT	FLT1	0.772476839
514	0.0000	0.641603797	0.388	0.125	0.0000	EVT	CLIC3	0.641603797
515	0.0000	0.649443397	0.65	0.348	0.0000	EVT	COTL1	0.649443397
516	0.0000	0.518358948	0.416	0.149	0.0000	EVT	CYP11A1	0.518358948
517	0.0000	0.588362794	0.509	0.221	0.0000	EVT	C12orf75	0.588362794
518	0.0000	0.596823835	0.678	0.375	0.0000	EVT	EPAS1	0.596823835
519	0.0000	0.733524606	0.762	0.591	0.0000	EVT	FN1	0.733524606
520	0.0000	0.525762138	0.502	0.218	0.0000	EVT	VASP	0.525762138
521	0.0000	0.49049207	0.441	0.164	0.0000	EVT	ATP11A	0.49049207
522	0.0000	0.548077755	0.48	0.21	0.0000	EVT	GPC1	0.548077755
523	0.0000	0.564770947	0.689	0.473	0.0000	EVT	LIMA1	0.564770947
524	0.0000	0.594447689	0.502	0.252	0.0000	EVT	BZW2	0.594447689
525	0.0000	0.582817647	0.72	0.542	0.0000	EVT	COL4A2	0.582817647
526	0.0000	0.614327769	0.619	0.4	0.0000	EVT	RALBP1	0.614327769

527	0.0000	0.478692886	0.445	0.191	0.0000	EVT	AFAP1	0.478692886
528	0.0000	0.537697383	0.623	0.298	0.0000	EVT	S100P	0.537697383
529	0.0000	0.751705164	0.522	0.264	0.0000	EVT	CSH1	0.751705164
530	0.0000	0.478897193	0.522	0.25	0.0000	EVT	EZR	0.478897193
531	0.0000	0.774180599	0.467	0.248	0.0000	EVT	H19	0.774180599
532	0.0000	0.652184416	0.498	0.281	0.0000	EVT	HSPG2	0.652184416
533	0.0000	0.543127122	0.518	0.308	0.0000	EVT	TCF7L2	0.543127122
534	0.0000	0.494098221	0.595	0.383	0.0000	EVT	TEAD1	0.494098221
535	0.0000	0.555061567	0.643	0.497	0.0000	EVT	TFPI	0.555061567
536	0.0000	0.515278975	0.577	0.397	0.0000	EVT	JPT1	0.515278975
537	0.0000	0.482858793	0.48	0.304	0.0000	EVT	CBLB	0.482858793
538	0.0000	1.395841794	0.806	0.236	0.0000	APCs & MACs	CD74	1.395841794
539	0.0000	1.358922954	0.722	0.163	0.0000	APCs & MACs	HLA-DRA	1.358922954
540	0.0000	1.221824315	0.708	0.118	0.0000	APCs & MACs	HLA-DRB1	1.221824315
541	0.0000	1.161376372	0.629	0.146	0.0000	APCs & MACs	HLA-DPA1	1.161376372
542	0.0000	1.096548224	0.588	0.056	0.0000	APCs & MACs	HLA-DQB1	1.096548224
543	0.0000	1.069779925	0.593	0.152	0.0000	APCs & MACs	HLA-DPB1	1.069779925
544	0.0000	1.052231115	0.499	0.07	0.0000	APCs & MACs	CD14	1.052231115
545	0.0000	1.018224393	0.557	0.025	0.0000	APCs & MACs	HLA-DQA1	1.018224393
546	0.0000	0.951736172	0.628	0.203	0.0000	APCs & MACs	CTSS	0.951736172
547	0.0000	0.886308077	0.529	0.082	0.0000	APCs & MACs	LAPTM5	0.886308077
548	0.0000	0.818116086	0.477	0.067	0.0000	APCs & MACs	CD37	0.818116086
549	0.0000	0.799121112	0.332	0.058	0.0000	APCs & MACs	C1QA	0.799121112
550	0.0000	0.78466512	0.426	0.043	0.0000	APCs & MACs	ADAP2	0.78466512
551	0.0000	0.731753799	0.35	0.024	0.0000	APCs & MACs	MRC1	0.731753799
552	0.0000	0.729323662	0.35	0.017	0.0000	APCs & MACs	CYBB	0.729323662
553	0.0000	0.71524002	0.576	0.136	0.0000	APCs & MACs	PTPRC	0.71524002
554	0.0000	0.714339013	0.305	0.043	0.0000	APCs & MACs	C1QB	0.714339013
555	0.0000	0.712841477	0.35	0.017	0.0000	APCs & MACs	CD163	0.712841477
556	0.0000	0.70724914	0.435	0.069	0.0000	APCs & MACs	HLA-DMA	0.70724914
557	0.0000	0.700574281	0.403	0.055	0.0000	APCs & MACs	ITGB2	0.700574281
558	0.0000	0.700157757	0.402	0.037	0.0000	APCs & MACs	CD83	0.700157757
559	0.0000	0.678232779	0.282	0.02	0.0000	APCs & MACs	C1QC	0.678232779
560	0.0000	0.672052203	0.322	0.029	0.0000	APCs & MACs	IGSF6	0.672052203
561	0.0000	0.651420726	0.406	0.043	0.0000	APCs & MACs	HLA-DMB	0.651420726
562	0.0000	0.64538228	0.395	0.044	0.0000	APCs & MACs	PLEK	0.64538228
563	0.0000	0.642254938	0.278	0.015	0.0000	APCs & MACs	CD163L1	0.642254938
564	0.0000	0.641363627	0.245	0.006	0.0000	APCs & MACs	MS4A1	0.641363627
565	0.0000	0.639735336	0.35	0.021	0.0000	APCs & MACs	CD84	0.639735336
566	0.0000	0.624633047	0.342	0.022	0.0000	APCs & MACs	CIITA	0.624633047
567	0.0000	0.620732741	0.327	0.016	0.0000	APCs & MACs	MPEG1	0.620732741
568	0.0000	0.617713257	0.384	0.037	0.0000	APCs & MACs	NCKAP1L	0.617713257
569	0.0000	0.611656121	0.353	0.033	0.0000	APCs & MACs	PHACTR1	0.611656121
570	0.0000	0.607744164	0.253	0.013	0.0000	APCs & MACs	MS4A6A	0.607744164

571	0.0000	0.586239699	0.305	0.022	0.0000	APCs & MACs	1-Mar	0.586239699
572	0.0000	0.571840609	0.177	0.009	0.0000	APCs & MACs	IGHM	0.571840609
573	0.0000	0.567598068	0.29	0.023	0.0000	APCs & MACs	SLCO2B1	0.567598068
574	0.0000	0.563143802	0.271	0.02	0.0000	APCs & MACs	AIF1	0.563143802
575	0.0000	0.555086256	0.328	0.027	0.0000	APCs & MACs	IRF8	0.555086256
576	0.0000	0.549614407	0.217	0.006	0.0000	APCs & MACs	CD79A	0.549614407
577	0.0000	0.547630307	0.288	0.024	0.0000	APCs & MACs	C5AR1	0.547630307
578	0.0000	0.545381018	0.252	0.009	0.0000	APCs & MACs	MS4A7	0.545381018
579	0.0000	0.544227776	0.312	0.009	0.0000	APCs & MACs	SPI1	0.544227776
580	0.0000	0.542950901	0.288	0.029	0.0000	APCs & MACs	FGL2	0.542950901
581	0.0000	0.538319094	0.372	0.062	0.0000	APCs & MACs	CD53	0.538319094
582	0.0000	0.52643499	0.329	0.025	0.0000	APCs & MACs	SYK	0.52643499
583	0.0000	0.520169068	0.311	0.03	0.0000	APCs & MACs	PRKCB	0.520169068
584	0.0000	0.49963126	0.248	0.018	0.0000	APCs & MACs	FCGR2A	0.49963126
585	0.0000	0.496239786	0.233	0.021	0.0000	APCs & MACs	LYZ	0.496239786
586	0.0000	0.491144598	0.316	0.034	0.0000	APCs & MACs	EVI2B	0.491144598
587	0.0000	0.486335486	0.229	0.023	0.0000	APCs & MACs	CSF1R	0.486335486
588	0.0000	0.462189033	0.252	0.025	0.0000	APCs & MACs	P2RX7	0.462189033
589	0.0000	0.449864486	0.251	0.006	0.0000	APCs & MACs	TFEC	0.449864486
590	0.0000	0.448864894	0.246	0.018	0.0000	APCs & MACs	NPL	0.448864894
591	0.0000	0.440854311	0.216	0.008	0.0000	APCs & MACs	MSR1	0.440854311
592	0.0000	0.623419903	0.373	0.077	0.0000	APCs & MACs	NRP2	0.623419903
593	0.0000	0.444188584	0.199	0.02	0.0000	APCs & MACs	CD79B	0.444188584
594	0.0000	0.494786679	0.324	0.057	0.0000	APCs & MACs	CD48	0.494786679
595	0.0000	0.552106937	0.314	0.055	0.0000	APCs & MACs	FCER1G	0.552106937
596	0.0000	0.643474082	0.367	0.075	0.0000	APCs & MACs	TYROBP	0.643474082
597	0.0000	0.708329719	0.331	0.069	0.0000	APCs & MACs	CXCL3	0.708329719
598	0.0000	0.609831536	0.41	0.1	0.0000	APCs & MACs	MEF2C	0.609831536
599	0.0000	0.601899774	0.264	0.043	0.0000	APCs & MACs	STAB1	0.601899774
600	0.0000	0.666474468	0.392	0.097	0.0000	APCs & MACs	CCL3	0.666474468
601	0.0000	0.835001359	0.614	0.314	0.0000	APCs & MACs	CTSZ	0.835001359
602	0.0000	0.680583001	0.192	0.025	0.0000	APCs & MACs	IGKC	0.680583001
603	0.0000	0.553473952	0.366	0.102	0.0000	APCs & MACs	RASSF4	0.553473952
604	0.0000	0.472751098	0.271	0.054	0.0000	APCs & MACs	BIRC3	0.472751098
605	0.0000	0.549213744	0.43	0.147	0.0000	APCs & MACs	RNASET2	0.549213744
606	0.0000	0.537015111	0.434	0.143	0.0000	APCs & MACs	ARHGDIB	0.537015111
607	0.0000	0.605100062	0.287	0.072	0.0000	APCs & MACs	CCL2	0.605100062
608	0.0000	0.443619543	0.331	0.087	0.0000	APCs & MACs	ITGAX	0.443619543
609	0.0000	0.579298144	0.305	0.085	0.0000	APCs & MACs	CXCL2	0.579298144
610	0.0000	0.439406021	0.298	0.083	0.0000	APCs & MACs	PLXNC1	0.439406021
611	0.0000	0.471410465	0.21	0.042	0.0000	APCs & MACs	LTB	0.471410465
612	0.0000	0.66289843	0.586	0.323	0.0000	APCs & MACs	ZEB2	0.66289843
613	0.0000	0.595466192	0.417	0.162	0.0000	APCs & MACs	SAMHD1	0.595466192
614	0.0000	0.464631068	0.312	0.09	0.0000	APCs & MACs	CXCR4	0.464631068

615	0.0000	0.501659811	0.269	0.071	0.0000	APCs & MACs	CD52	0.501659811
616	0.0000	0.441825553	0.312	0.1	0.0000	APCs & MACs	KIAA0930	0.441825553
617	0.0000	0.437497363	0.354	0.121	0.0000	APCs & MACs	MBP	0.437497363
618	0.0000	0.567065944	0.354	0.123	0.0000	APCs & MACs	CCL4	0.567065944
619	0.0000	0.436070701	0.363	0.125	0.0000	APCs & MACs	POU2F2	0.436070701
620	0.0000	0.441776824	0.375	0.141	0.0000	APCs & MACs	CELF2	0.441776824
621	0.0000	0.469720417	0.358	0.137	0.0000	APCs & MACs	ARHGAP18	0.469720417
622	0.0000	0.442017387	0.366	0.134	0.0000	APCs & MACs	FYB1	0.442017387
623	0.0000	0.45441824	0.378	0.151	0.0000	APCs & MACs	CAPG	0.45441824
624	0.0000	0.550580746	0.444	0.224	0.0000	APCs & MACs	ITPR2	0.550580746
625	0.0000	0.505571722	0.423	0.184	0.0000	APCs & MACs	SRGN	0.505571722
626	0.0000	0.458150087	0.689	0.51	0.0000	APCs & MACs	ANKRD12	0.458150087
627	0.0000	0.451463682	0.666	0.506	0.0000	APCs & MACs	CYBA	0.451463682
628	0.0000	0.5237104	0.592	0.424	0.0000	APCs & MACs	ASAH1	0.5237104
629	0.0000	0.53811391	0.362	0.17	0.0000	APCs & MACs	LGMN	0.53811391
630	0.0000	0.470035333	0.347	0.159	0.0000	APCs & MACs	FNIP2	0.470035333
631	0.0000	0.471656414	0.332	0.154	0.0000	APCs & MACs	MAFB	0.471656414
632	0.0000	0.473885908	0.403	0.213	0.0000	APCs & MACs	REL	0.473885908
633	0.0000	0.438366755	0.373	0.186	0.0000	APCs & MACs	BMP2K	0.438366755
634	0.0000	0.448370593	0.449	0.264	0.0000	APCs & MACs	STX7	0.448370593
635	0.0000	0.460337787	0.372	0.195	0.0000	APCs & MACs	DMXL2	0.460337787
636	0.0000	0.438740535	0.455	0.321	0.0000	APCs & MACs	CCDC88A	0.438740535
637	0.0000	0.448760296	0.489	0.42	0.0000	APCs & MACs	PLD3	0.448760296
638	0.0000	1.640911699	0.831	0.03	0.0000	EC	VWF	1.640911699
639	0.0000	1.531227496	0.824	0.036	0.0000	EC	PCDH17	1.531227496
640	0.0000	1.511922468	0.901	0.038	0.0000	EC	EGFL7	1.511922468
641	0.0000	1.420787201	0.868	0.061	0.0000	EC	PECAM1	1.420787201
642	0.0000	1.365743286	0.803	0.033	0.0000	EC	ADGRL4	1.365743286
643	0.0000	1.340306853	0.795	0.038	0.0000	EC	CD93	1.340306853
644	0.0000	1.333841277	0.858	0.325	0.0000	EC	A2M	1.333841277
645	0.0000	1.31549373	0.784	0.064	0.0000	EC	SOX18	1.31549373
646	0.0000	1.302784072	0.774	0.026	0.0000	EC	CD34	1.302784072
647	0.0000	1.291816571	0.713	0.086	0.0000	EC	ID1	1.291816571
648	0.0000	1.286898555	0.739	0.033	0.0000	EC	CLDN5	1.286898555
649	0.0000	1.280565362	0.864	0.088	0.0000	EC	ARHGAP29	1.280565362
650	0.0000	1.247041908	0.79	0.127	0.0000	EC	CALCRL	1.247041908
651	0.0000	1.218835579	0.694	0.049	0.0000	EC	RNASE1	1.218835579
652	0.0000	1.178944398	0.762	0.077	0.0000	EC	CRIP2	1.178944398
653	0.0000	1.164528032	0.785	0.118	0.0000	EC	TM4SF1	1.164528032
654	0.0000	1.161465083	0.711	0.017	0.0000	EC	EMCN	1.161465083
655	0.0000	1.139546207	0.922	0.372	0.0000	EC	TCF4	1.139546207
656	0.0000	1.132759942	0.735	0.062	0.0000	EC	PODXL	1.132759942
657	0.0000	1.129525769	0.713	0.018	0.0000	EC	CLEC14A	1.129525769
658	0.0000	1.114978029	0.728	0.159	0.0000	EC	ID3	1.114978029

659	0.0000	1.114963817	0.738	0.17	0.0000	EC	HYAL2	1.114963817
660	0.0000	1.108890301	0.529	0.024	0.0000	EC	ACKR1	1.108890301
661	0.0000	1.094850077	0.664	0.123	0.0000	EC	RAMP2	1.094850077
662	0.0000	1.094427721	0.788	0.101	0.0000	EC	RALGAPA2	1.094427721
663	0.0000	1.084984627	0.838	0.353	0.0000	EC	IFI27	1.084984627
664	0.0000	1.040837683	0.683	0.113	0.0000	EC	KLF2	1.040837683
665	0.0000	1.031049383	0.773	0.159	0.0000	EC	ITGA6	1.031049383
666	0.0000	1.030965285	0.537	0.029	0.0000	EC	MMRN1	1.030965285
667	0.0000	1.024345338	0.536	0.02	0.0000	EC	AQP1	1.024345338
668	0.0000	1.018178243	0.738	0.024	0.0000	EC	SHANK3	1.018178243
669	0.0000	1.01505923	0.647	0.012	0.0000	EC	TM4SF18	1.01505923
670	0.0000	1.009791788	0.729	0.136	0.0000	EC	ADAM15	1.009791788
671	0.0000	1.008012064	0.686	0.018	0.0000	EC	PTPRB	1.008012064
672	0.0000	0.997907071	0.656	0.011	0.0000	EC	MMRN2	0.997907071
673	0.0000	0.993697418	0.744	0.141	0.0000	EC	NPDC1	0.993697418
674	0.0000	0.987128809	0.665	0.037	0.0000	EC	LMO2	0.987128809
675	0.0000	0.98678444	0.614	0.032	0.0000	EC	FAM167B	0.98678444
676	0.0000	0.983570218	0.683	0.074	0.0000	EC	LDB2	0.983570218
677	0.0000	0.973924317	0.668	0.028	0.0000	EC	ADGRF5	0.973924317
678	0.0000	0.973349421	0.642	0.013	0.0000	EC	ECSCR	0.973349421
679	0.0000	0.971713751	0.674	0.054	0.0000	EC	HOXD9	0.971713751
680	0.0000	0.96775169	0.596	0.052	0.0000	EC	NUAK1	0.96775169
681	0.0000	0.964623473	0.605	0.033	0.0000	EC	RAMP3	0.964623473
682	0.0000	0.961385987	0.731	0.14	0.0000	EC	MCAM	0.961385987
683	0.0000	0.953831123	0.7	0.078	0.0000	EC	SMAD1	0.953831123
684	0.0000	0.9506334	0.683	0.015	0.0000	EC	TIE1	0.9506334
685	0.0000	0.948576709	0.705	0.053	0.0000	EC	CDH5	0.948576709
686	0.0000	0.938518311	0.644	0.01	0.0000	EC	CXorf36	0.938518311
687	0.0000	0.93780753	0.652	0.037	0.0000	EC	ESAM	0.93780753
688	0.0000	0.936014003	0.814	0.317	0.0000	EC	ENG	0.936014003
689	0.0000	0.93440141	0.656	0.019	0.0000	EC	ADCY4	0.93440141
690	0.0000	0.929495929	0.733	0.094	0.0000	EC	MEF2C	0.929495929
691	0.0000	0.926555483	0.645	0.055	0.0000	EC	KDR	0.926555483
692	0.0000	0.921253471	0.637	0.018	0.0000	EC	RAPGEF5	0.921253471
693	0.0000	0.915421708	0.783	0.266	0.0000	EC	HSPG2	0.915421708
694	0.0000	0.903040417	0.74	0.21	0.0000	EC	ARRDC3	0.903040417
695	0.0000	0.899924035	0.624	0.017	0.0000	EC	CYYR1	0.899924035
696	0.0000	0.891038831	0.723	0.178	0.0000	EC	NFIB	0.891038831
697	0.0000	0.888455451	0.762	0.205	0.0000	EC	KIAA0355	0.888455451
698	0.0000	0.876596616	0.762	0.16	0.0000	EC	FLT1	0.876596616
699	0.0000	0.867934714	0.622	0.012	0.0000	EC	PCAT19	0.867934714
700	0.0000	0.861259092	0.665	0.155	0.0000	EC	HEG1	0.861259092
701	0.0000	0.855585221	0.632	0.055	0.0000	EC	ICAM2	0.855585221
702	0.0000	0.847337065	0.614	0.009	0.0000	EC	ROBO4	0.847337065

703	0.0000	0.843269965	0.698	0.122	0.0000	EC	MAGI1	0.843269965
704	0.0000	0.843116774	0.601	0.071	0.0000	EC	LEPR	0.843116774
705	0.0000	0.838385834	0.66	0.133	0.0000	EC	HLA-DRB1	0.838385834
706	0.0000	0.824341899	0.601	0.036	0.0000	EC	NOSTRIN	0.824341899
707	0.0000	0.822451497	0.641	0.135	0.0000	EC	SSFA2	0.822451497
708	0.0000	0.821113487	0.587	0.025	0.0000	EC	ZNF385D	0.821113487
709	0.0000	0.808734448	0.537	0.037	0.0000	EC	THBD	0.808734448
710	0.0000	0.801228567	0.576	0.048	0.0000	EC	ENTPD1	0.801228567
711	0.0000	0.793785602	0.611	0.02	0.0000	EC	RHOJ	0.793785602
712	0.0000	0.792628731	0.629	0.083	0.0000	EC	MGST2	0.792628731
713	0.0000	0.787001356	0.541	0.042	0.0000	EC	IL33	0.787001356
714	0.0000	0.786567576	0.555	0.019	0.0000	EC	PLCB4	0.786567576
715	0.0000	0.78477105	0.596	0.009	0.0000	EC	ERG	0.78477105
716	0.0000	0.78387916	0.533	0.019	0.0000	EC	NOTCH4	0.78387916
717	0.0000	0.771624663	0.588	0.034	0.0000	EC	SH2D3C	0.771624663
718	0.0000	0.761722876	0.557	0.026	0.0000	EC	ABCB1	0.761722876
719	0.0000	0.756258329	0.565	0.008	0.0000	EC	SHE	0.756258329
720	0.0000	0.752091165	0.639	0.104	0.0000	EC	LAMA5	0.752091165
721	0.0000	0.750539758	0.517	0.006	0.0000	EC	ADAMTSL1	0.750539758
722	0.0000	0.812990449	0.684	0.199	0.0000	EC	SLC39A10	0.812990449
723	0.0000	0.869944966	0.739	0.227	0.0000	EC	ADAMTS9	0.869944966
724	0.0000	0.809842304	0.759	0.231	0.0000	EC	ELK3	0.809842304
725	0.0000	0.853938303	0.939	0.498	0.0000	EC	SPTBN1	0.853938303
726	0.0000	0.847425051	0.718	0.226	0.0000	EC	UACA	0.847425051
727	0.0000	0.796316089	0.747	0.223	0.0000	EC	PALMD	0.796316089
728	0.0000	0.855856099	0.854	0.371	0.0000	EC	NFAT5	0.855856099
729	0.0000	0.755736639	0.664	0.18	0.0000	EC	SRGN	0.755736639
730	0.0000	0.80826229	0.728	0.268	0.0000	EC	TSHZ2	0.80826229
731	0.0000	0.808757132	0.65	0.213	0.0000	EC	SESN3	0.808757132
732	0.0000	0.910234533	0.735	0.252	0.0000	EC	CD74	0.910234533
733	0.0000	0.772745116	0.72	0.266	0.0000	EC	UGCG	0.772745116
734	0.0000	0.752956247	0.629	0.18	0.0000	EC	HLA-DRA	0.752956247
735	0.0000	0.751348595	0.759	0.327	0.0000	EC	TSC22D1	0.751348595
736	0.0000	0.772851445	0.752	0.36	0.0000	EC	GNG11	0.772851445
737	0.0000	0.769185972	0.841	0.417	0.0000	EC	SPARCL1	0.769185972
738	0.0000	1.58720082	0.825	0.113	0.0000	EEC	SLPI	1.58720082
739	0.0000	1.556701725	0.905	0.294	0.0000	EEC	GPX3	1.556701725
740	0.0000	1.47632594	0.764	0.102	0.0000	EEC	CP	1.47632594
741	0.0000	1.406070658	0.982	0.695	0.0000	EEC	PAEP	1.406070658
742	0.0000	1.386657703	0.727	0.124	0.0000	EEC	DEFB1	1.386657703
743	0.0000	1.384999396	0.728	0.148	0.0000	EEC	RIMKLB	1.384999396
744	0.0000	1.326721149	0.704	0.04	0.0000	EEC	IRX3	1.326721149
745	0.0000	1.311539028	0.712	0.047	0.0000	EEC	SLC18A2	1.311539028
746	0.0000	1.253611123	0.65	0.018	0.0000	EEC	LINC01541	1.253611123

747	0.0000	1.243320331	0.71	0.041	0.0000	EEC	C2CD4B	1.243320331
748	0.0000	1.23049739	0.751	0.199	0.0000	EEC	SLC7A2	1.23049739
749	0.0000	1.199346467	0.655	0.097	0.0000	EEC	MT1G	1.199346467
750	0.0000	1.187662814	0.635	0.041	0.0000	EEC	LINC01502	1.187662814
751	0.0000	1.186943767	0.686	0.029	0.0000	EEC	PAX8	1.186943767
752	0.0000	1.135244939	0.625	0.048	0.0000	EEC	AGR2	1.135244939
753	0.0000	1.118954039	0.599	0.055	0.0000	EEC	SCGB2A1	1.118954039
754	0.0000	1.117993435	0.64	0.039	0.0000	EEC	MUC16	1.117993435
755	0.0000	1.094981349	0.627	0.03	0.0000	EEC	TSPAN1	1.094981349
756	0.0000	1.093756957	0.671	0.045	0.0000	EEC	TC2N	1.093756957
757	0.0000	1.089084169	0.661	0.148	0.0000	EEC	PLA2G16	1.089084169
758	0.0000	1.068718087	0.664	0.079	0.0000	EEC	EPCAM	1.068718087
759	0.0000	1.062565691	0.619	0.047	0.0000	EEC	CLDN3	1.062565691
760	0.0000	1.026194628	0.594	0.03	0.0000	EEC	C2CD4A	1.026194628
761	0.0000	1.021028587	0.638	0.067	0.0000	EEC	SLC1A1	1.021028587
762	0.0000	1.016250135	0.689	0.16	0.0000	EEC	BACE2	1.016250135
763	0.0000	1.013142357	0.664	0.06	0.0000	EEC	UCA1	1.013142357
764	0.0000	0.984647197	0.54	0.02	0.0000	EEC	WFDC2	0.984647197
765	0.0000	0.977750554	0.669	0.128	0.0000	EEC	TM4SF1	0.977750554
766	0.0000	0.941244568	0.589	0.045	0.0000	EEC	MUC1	0.941244568
767	0.0000	0.923965827	0.571	0.019	0.0000	EEC	PDZK1IP1	0.923965827
768	0.0000	0.923485504	0.594	0.042	0.0000	EEC	LAMB3	0.923485504
769	0.0000	0.911503757	0.658	0.132	0.0000	EEC	SPINT2	0.911503757
770	0.0000	0.898040957	0.532	0.048	0.0000	EEC	MT1F	0.898040957
771	0.0000	0.89338117	0.579	0.061	0.0000	EEC	DEPTOR	0.89338117
772	0.0000	0.877019335	0.584	0.02	0.0000	EEC	GABRP	0.877019335
773	0.0000	0.870976875	0.588	0.014	0.0000	EEC	SLC15A1	0.870976875
774	0.0000	0.8656649	0.511	0.019	0.0000	EEC	DRAIC	0.8656649
775	0.0000	0.843884336	0.581	0.076	0.0000	EEC	TACSTD2	0.843884336
776	0.0000	0.83725888	0.483	0.023	0.0000	EEC	FXYD2	0.83725888
777	0.0000	0.824246707	0.619	0.096	0.0000	EEC	SLC6A6	0.824246707
778	0.0000	0.822424006	0.519	0.012	0.0000	EEC	PTPRR	0.822424006
779	0.0000	0.816303975	0.614	0.095	0.0000	EEC	CDH1	0.816303975
780	0.0000	0.813482919	0.547	0.047	0.0000	EEC	CAPS	0.813482919
781	0.0000	0.802420993	0.535	0.02	0.0000	EEC	ATP2C2	0.802420993
782	0.0000	0.791024072	0.493	0.018	0.0000	EEC	KIAA1324	0.791024072
783	0.0000	0.775078331	0.46	0.018	0.0000	EEC	LCN2	0.775078331
784	0.0000	0.774628937	0.463	0.026	0.0000	EEC	COMP	0.774628937
785	0.0000	0.769981289	0.47	0.015	0.0000	EEC	TSPAN8	0.769981289
786	0.0000	0.763649933	0.534	0.018	0.0000	EEC	PHYHIPL	0.763649933
787	0.0000	0.753332907	0.545	0.051	0.0000	EEC	DMKN	0.753332907
788	0.0000	0.752104435	0.381	0.031	0.0000	EEC	GAST	0.752104435
789	0.0000	0.733018028	0.434	0.015	0.0000	EEC	AOC1	0.733018028
790	0.0000	0.730330283	0.574	0.072	0.0000	EEC	SLC25A29	0.730330283

791	0.0000	0.720738229	0.45	0.012	0.0000	EEC	C4BPA	0.720738229
792	0.0000	0.717318277	0.525	0.029	0.0000	EEC	FAM84B	0.717318277
793	0.0000	0.714750135	0.524	0.05	0.0000	EEC	CA12	0.714750135
794	0.0000	0.702924243	0.491	0.01	0.0000	EEC	SMIM22	0.702924243
795	0.0000	0.690882958	0.48	0.035	0.0000	EEC	ARG2	0.690882958
796	0.0000	0.688350322	0.465	0.038	0.0000	EEC	FXYD3	0.688350322
797	0.0000	0.676556963	0.522	0.048	0.0000	EEC	PRUNE2	0.676556963
798	0.0000	0.676231549	0.388	0.02	0.0000	EEC	RBP4	0.676231549
799	0.0000	0.664431542	0.426	0.007	0.0000	EEC	ADGRF1	0.664431542
800	0.0000	0.664251833	0.507	0.057	0.0000	EEC	ISG20	0.664251833
801	0.0000	1.051279124	0.71	0.173	0.0000	EEC	PERP	1.051279124
802	0.0000	1.165934272	0.664	0.16	0.0000	EEC	PCCA	1.165934272
803	0.0000	0.83277159	0.635	0.122	0.0000	EEC	CLDN4	0.83277159
804	0.0000	1.263562341	0.823	0.298	0.0000	EEC	ACSL4	1.263562341
805	0.0000	0.681361647	0.588	0.104	0.0000	EEC	ARHGAP29	0.681361647
806	0.0000	1.373040114	0.818	0.295	0.0000	EEC	CXCL14	1.373040114
807	0.0000	1.115743239	0.836	0.311	0.0000	EEC	CD24	1.115743239
808	0.0000	0.97818372	0.684	0.177	0.0000	EEC	IVNS1ABP	0.97818372
809	0.0000	1.030009728	0.645	0.166	0.0000	EEC	MAOA	1.030009728
810	0.0000	0.840556129	0.642	0.15	0.0000	EEC	RNASET2	0.840556129
811	0.0000	0.676941418	0.586	0.114	0.0000	EEC	JUP	0.676941418
812	0.0000	0.892628575	0.606	0.151	0.0000	EEC	MTF1	0.892628575
813	0.0000	0.856701687	0.633	0.172	0.0000	EEC	PKDCC	0.856701687
814	0.0000	0.789398017	0.596	0.139	0.0000	EEC	CD9	0.789398017
815	0.0000	1.041669927	0.709	0.24	0.0000	EEC	SLC44A1	1.041669927
816	0.0000	1.092142127	0.766	0.323	0.0000	EEC	ANXA4	1.092142127
817	0.0000	0.892124624	0.655	0.196	0.0000	EEC	MT1E	0.892124624
818	0.0000	0.986124631	0.809	0.388	0.0000	EEC	GADD45A	0.986124631
819	0.0000	0.798515849	0.668	0.214	0.0000	EEC	C12orf75	0.798515849
820	0.0000	0.8983077	0.627	0.199	0.0000	EEC	SEC11C	0.8983077
821	0.0000	0.940112284	0.681	0.252	0.0000	EEC	ATP6V1A	0.940112284
822	0.0000	0.806465207	0.622	0.197	0.0000	EEC	SCCPDH	0.806465207
823	0.0000	0.949208092	0.83	0.506	0.0000	EEC	IL6ST	0.949208092
824	0.0000	0.719532093	0.584	0.18	0.0000	EEC	MRPS2	0.719532093
825	0.0000	0.726467976	0.558	0.183	0.0000	EEC	EPHX1	0.726467976
826	0.0000	0.913876906	0.71	0.323	0.0000	EEC	CLU	0.913876906
827	0.0000	0.818551563	0.881	0.614	0.0000	EEC	FBLN1	0.818551563
828	0.0000	0.860904758	0.761	0.426	0.0000	EEC	CD55	0.860904758
829	0.0000	0.685327732	0.643	0.246	0.0000	EEC	CNDP2	0.685327732
830	0.0000	0.757753186	0.761	0.33	0.0000	EEC	KRT19	0.757753186
831	0.0000	0.795043563	0.763	0.345	0.0000	EEC	SYNE2	0.795043563
832	0.0000	0.677554193	0.66	0.235	0.0000	EEC	KRT7	0.677554193
833	0.0000	0.76412271	0.73	0.418	0.0000	EEC	ARID5B	0.76412271
834	0.0000	0.715321651	0.676	0.314	0.0000	EEC	DDX52	0.715321651

835	0.0000	0.703619293	0.632	0.31	0.0000	EEC	FAM177A1	0.703619293
836	0.0000	0.666211124	0.635	0.263	0.0000	EEC	DPP4	0.666211124
837	0.0000	0.732443515	0.651	0.299	0.0000	EEC	CRYAB	0.732443515

Table S3, TOP 100 genes for clustering single-cell data, Related to Figure 3B

Sample	VCT	FB 1	FB 2	SCT	T & NK	EVT	APCs & MACs	EC	EEC	Total
PV	1219	2375	3287	1369	1042	252	686	121	113	10464
PVOs	519	1796	3564	1392	1137	202	578	676	498	10362

Table S4-1, Cells per sample in each cluster Related to Figure 3B

Sample	MAC	TC	NK1	NK2	APC	Total
PV	568	503	240	287	112	1710
PVOs	324	248	498	339	240	1649

Table S4-2, Immune cell per sample in each cluster, Related to Figure 3F

Sample	CD45+	CD3+	T %	CD14+	MACs %	CD56+	NK %	Other cells	OC %
PV1	5807	22	0.38%	686	11.81%	4031	69.42%	1068	18.39%
PVOs-1	2204	520	23.59%	179	8.12%	1171	53.13%	334	15.15%
PV2	6363	716	11.25%	690	10.84%	4250	66.79%	707	11.11%
PVOs-2	7314	435	5.95%	607	8.30%	4827	66.00%	1445	19.76%
PV3	7102	943	13.28%	379	5.34%	4307	60.64%	1473	20.74%
PVOs-3	5286	1024	19.37%	169	3.20%	3160	59.78%	933	17.65%
PV4	12279	1031	8.40%	2194	17.87%	7437	60.57%	1617	13.17%
PVOs-4	3581	806	22.51%	227	6.34%	1799	50.24%	749	20.92%

Table S5, Flow cytometry analysis to identify immune components in placenta villi and their derived PVOs at d7, Related to Figure 3 H

Exciting light	Antibody	Fluorescein	Company	Product Number
405nm	CD56	BV421	BD	562751
	CD3	BV510	BD	563109
	EpCAM	BV605	BD	563182
	CD16	BV711	BD	563127
488nm	CD14	FITC	Biolegend	325604
	CD163	PE	Biolegend	333606
	CD8	Percp-cy5.5	BD	560662
	CD4	PE-CY7	BD	557852
633nm	CD86	APC	Biolegend	305412
	LIVE/DEAD	AF700	BD	564997
	CD45	APC-Cy7	BD	557833

Table S6, Antibodies of immune cell analysis panel Related to Figure S3A
